# Muscle weakness but also contractures contribute to the progressive gait pathology in children with Duchenne muscular dystrophy: a simulation study

**DOI:** 10.1186/s12984-025-01631-x

**Published:** 2025-05-04

**Authors:** Ines Vandekerckhove, Lars D’Hondt, Dhruv Gupta, Bram Van Den Bosch, Marleen Van den Hauwe, Nathalie Goemans, Liesbeth De Waele, Anja Van Campenhout, Kaat Desloovere, Friedl De Groote

**Affiliations:** 1 Department of Rehabilitation Sciences, KU Leuven, Leuven, Belgium; 2 Department of Movement Sciences, KU Leuven, Leuven, Belgium; 3 Department of Child Neurology, University Hospital Leuven, Leuven, Belgium; 4 Department of Development and Regeneration, KU Leuven, Leuven, Belgium; 5 Department of Orthopedics, University Hospital Leuven, Leuven, Belgium; 6 Clinical Motion Analysis Laboratory, University Hospital Leuven, Pellenberg, Belgium

**Keywords:** Duchenne muscular dystrophy, Muscle weakness, Contractures, Musculoskeletal modeling, Predictive simulations

## Abstract

**Background:**

Muscle weakness and contractures cause gait deficits in children with Duchenne muscular dystrophy (DMD) but their relative contributions are poorly understood and hence it is unclear whether contractures should be treated. Therefore, we aimed to differentiate the effect of muscle weakness in isolation from weakness and contractures combined on the gait patterns.

**Methods:**

We used computer simulations that generate gait patterns based on a musculoskeletal model (without relying on experimental data) to establish the relationship between muscle impairments and gait deviations. We previously collected a longitudinal database of 137 repeated measurements in 30 boys with DMD and found that the data measured through 3D gait analysis could be clustered in three gait patterns. We estimated weakness based on data from fixed dynamometry, and contractures based on goniometry and clinical measures. Foot deformities were modeled by reducing the height of all foot segments and decreasing the strength of the intrinsic foot muscles. We created musculoskeletal models that either represented (1) the mean weakness; (2) the mean weakness and contractures; or (3) the mean weakness, contractures and foot deformities, in each gait pattern.

**Results:**

Simulations based on models with both weakness and contractures captured most (but not all) experimentally observed gait deviations, demonstrating the validity of our approach. While muscle weakness was primarily responsible for gait deviations, muscle contractures and foot deformities further contributed to gait deviations. Interestingly, the simulations predict that the combination of increasing weakness and contractures rather than increasing weakness alone causes loss of ambulation for the most affected gait pattern.

**Conclusions:**

Predictive simulations have the potential to elucidate causal relationships between muscle impairments and gait deviations in boys with DMD. In the future, they could be used to design targeted interventions (e.g. stretching, assistive devices) to prolong ambulation.

**Supplementary Information:**

The online version contains supplementary material available at 10.1186/s12984-025-01631-x.

## Background

Duchenne muscular dystrophy is a severe X-linked neuromuscular disorder caused by mutations in the *dystrophin* gene, leading to progressive muscle degeneration characterized by loss of contractile tissue and its replacement by fat and fibrotic tissue [1–3]. Subsequently, progressive muscle weakness, stiffness and contractures manifest, altering posture and gait, with loss of ambulation between 7.1 and 18.6 years of age (mean age: 12.7 years) [1, 4, 5]. Since there is no cure, rehabilitation, orthopedic, and orthotic treatments target muscle stiffness and contractures in order to prolong ambulation and slow disease progression [1]. However, conflicting results of orthopedic and orthotic treatments on gait decline have been reported [6–10]. Additionally, disease-modifying treatments show promise in further delaying the loss of ambulation and slowing disease progression. However, progress and clinical implementation have been hampered by the lack of sensitive outcome measures [11–14]. Improved understanding of how impairments contribute to gait pathology is necessary to enhance clinical decision-making, advance current rehabilitation, orthopedic and orthotic treatments, and support the development of novel treatments. Yet, insights into the causal relationships between underlying weakness and contractures, and gait pathology in DMD are still limited.

It is poorly understood how muscle weakness and stiffness interact in progressive DMD gait pathology. Although muscle weakness is considered the most important contributor to pathological gait in DMD [1, 5], the role of contractures in conjunction with weakness remains unclear. It has been postulated that boys with DMD could use the passive forces caused by contractures to compensate for muscle weakness [15], but also that increased contractures could lead to loss of ambulation if they undermine successful compensation mechanisms for muscle weakness [1]. We recently provided objective evidence that muscle weakness and contractures are associated with, and interact longitudinally with, gait pathology in DMD [16]. However, the co-occurrence and simultaneous decline of multiple impairments hamper disentangling the causal contribution of weakness from that of contractures to gait pathology in DMD based on experimental data alone.

Three gait patterns have been identified in growing children with DMD: a mildly affected gait pattern, a tiptoeing gait pattern, and a flexion gait pattern with distinct anterior pelvic tilt and posterior trunk leaning [17]. Both the tiptoeing and flexion gait patterns deviated more severely from the typically developing (TD) gait pattern than the mildly affected gait pattern, but they differed in the location (proximal versus distal) of the most severe deviations. The tiptoeing gait pattern exhibited the most severe deviations around the ankle, while the flexion gait pattern exhibited the most severe deviations around the proximal joints. Notably, abnormally large midfoot motion (i.e. midfoot break) was observed in boys with DMD exhibiting the flexion gait pattern.

Physics-based predictive simulations of gait are a powerful tool to unravel complex causal relationships between underlying impairments and pathological gait patterns. Such simulations generate novel movement patterns based on a musculoskeletal model (i.e., a mathematical description of the musculoskeletal system) and the assumption that gait is achieved by optimizing performance, without relying on experimental data [18, 19]. They therefore enable computation of the direct effect of a change in the musculoskeletal system on the gait pattern by adjusting model parameters, allowing to study both the isolated effects as well as interaction effects between underlying impairments on the gait pattern. Falisse et al. [20] improved the computational efficiency of forward simulations of 3D gait [20–23] allowing us to perform many simulations with complex 3D models [24]. D’Hondt et al.[23, 25] demonstrated that incorporating a dynamic three-segment foot model with flexible longitudinal arch into a complex 3D model (based on OpenSim’s gait2392 model) improved gait simulations and especially the agreement between simulated and experimentally observed ankle–foot kinematics. In addition, the more detailed foot model allows for modeling foot deformities. Since boys with DMD exhibit severe gait deviations affecting all planes and could present a midfoot break, complex 3D models are necessary to accurately simulate DMD gait.

Previous predictive simulations studies have examined the effects of weakness and/or stiffness on gait, but the interaction between these impairments has thus far only been investigated for the ankle. Ong et al. [26] showed that the isolated effect of severe plantar flexor weakness resulted in calcaneal gait without crouch, while the isolated effect of severe plantar flexor contracture resulted in equinus gait. Waterval et al. [27] found that a simulated gait pattern with 80% of plantar flexion weakness closely matched the experimental hip and ankle kinematics and kinetics of participants with bilateral plantar flexion weakness, thereby validating their predictive simulations. By investigating the isolated effect of incrementing plantar flexion weakness, they observed that pathological gait features particularly emerged when bilateral plantar flexion weakness exceeded 60%. However, these studies employed 2D musculoskeletal models, whereas gait deviations in DMD also occur in the frontal plane. In contrast, Falisse et al. [20] performed predictive simulations with complex 3D models and showed that increased hip weakness caused increased trunk sway, increased step width and decreased hip moments, and that increased plantar flexion weakness caused increased knee flexion, increased ankle dorsiflexion and decreased stride lengths that reduced ankle moments. Previous studies have thus been limited to examining the isolated effects of weakness or contracture in separate muscle groups and have not yet investigated the interaction between weakness and contractures on the gait pattern, except for the ankle. Additionally, there is still a need to evaluate whether predictive simulations of 3D gait could capture the key features of DMD gait and whether modeling increasing levels of impairments would result in the different DMD gait patterns, based on severity and location of the impairments [17].

The first aim of this study was to investigate the ability to simulate DMD gait using models that reflect the DMD-specific weakness and contractures. Specifically, we aimed to evaluate whether we can capture the mildly affected gait pattern, the tiptoeing gait pattern, and the flexion gait pattern [17] by modeling the experimentally measured muscle weakness and contractures of the children who exhibit these gait patterns. Given that the interaction between weakness and contractures is only poorly understood in DMD, the second aim of this study was to differentiate the effect of weakness alone from the effect of weakness in combination with contractures on the simulated gait patterns. Lastly, the third aim of this study was to investigate the effect of progressive impairments on the pathological gait pattern by modeling increasing levels of impairment severity.

## Methods

We performed a simulation study based on our previously collected longitudinal database[17, 28, 29].

### Experimental data

We used experimental data to create models that reflect the muscle impairments in children with DMD and to evaluate how accurately the simulations (see "Personalizing musculoskeletal model" section) capture the gait deviations.

Our collected longitudinal dataset consists of instrumented strength assessments, clinical examinations, and 3D gait analyses of 30 boys with DMD. These boys were repeatedly measured between 2015 and 2022 at multiple time points (median number of repeated sessions = 4; range number of repeated sessions = 1–10; mean time between repeated sessions = 0.74 ± 0.49 years) at the Clinical Motion Analysis Laboratory in the University Hospital Leuven campus Pellenberg, resulting in a dataset of 137 sessions. The data and collection procedures have been extensively described in our previous work [17, 28, 29].

Data collection was approved by the local ethics committee (Ethical Committee UZ Leuven/KU Leuven; S61324) under the Declaration of Helsinki. The parents or participants’ caregivers provided written informed consents. Participants aged 12 years or older provided informed assents. All methodology adhered to the relevant regulations and guidelines.

Body mass, height, and lower limb segment lengths were measured at each observation.

Hip extension, flexion and abduction, knee extension and flexion, and ankle plantar flexion and dorsiflexion muscle weakness were assessed with an instrumented strength assessment [30, 31]. To avoid fatigue and ensure participant cooperation, the instrumented strength assessment was performed unilaterally. If asymmetry was detected on the manual muscle testing during the clinical exam (not common), the weakest side was assessed. If muscle weakness was symmetrical, the assessed side was randomly chosen by flipping a coin. For each child, the same leg was assessed consistently across all longitudinal assessments. Participants performed maximal voluntary isometric contractions (MVIC) in standardized test positions on a fixed dynamometer (MicroFet, Hogan Health Industries, West Jordan, UT United States). The mean maximal joint torque per muscle group was calculated by multiplying the mean maximal force over one to three representative MVIC trials with its lever arm with respect to the joint. The strength outcomes of the children with DMD were expressed as percentages of the mean strength of TD children with a similar body mass and height (based on a dataset of 153 children [32]), reflecting strength deficits.

At each observation a standardized clinical examination was performed. Passive range of motion (ROM) of hip extension (modified Thomas test [33]), hip adduction (with extended hip and knee of the assessed leg and hip and knee flexed in 90° of the contralateral leg), the hamstrings (true popliteal angle [33]), and ankle dorsiflexion (with knee extended and knee flexed in 90° [33]) was assessed using goniometry. The age-related normative values of Mudge et al. [33] and Sankar et al. [34] were used as the reference. Differences between the ROM measurements of the children with DMD and the reference ROM measurements of TD children with a similar age were calculated, reflecting ROM deficits. Muscle stiffness of hip flexors, hip abductors, rectus femoris, hamstrings, plantar flexors (with knee extended and flexed) was also measured using a clinical stiffness scale during passive elongation (0–3 point ordinal scale: 0 = no increased resistance; 1 = minimal increased resistance at the end of ROM; 2 = increased resistance; 3 = highly pronounced resistance). This scale was inspired by the Ashworth scale to assess joint hyper-resistance in neurological diseases for which validity and reliability have been established [35, 36]. Given that there is no contribution from abnormal tone to increased resistance in DMD, we removed references to neurally induced increases in resistance such as ‘catch’. Both sides were assessed, but only the values from the side measured with the instrumented strength assessment were included for further analysis. Weakness of the abdominal and back muscles was evaluated with the manual muscle testing [37]. Strength of the abdominal and back muscles was converted to strength deficits, expressed as a percentage relative to the maximal score of 5.

Gait was measured by 3D gait analysis. The boys with DMD were instrumented with reflective markers (Plug-In Gait Full-body marker model with simple foot model (two foot markers); marker diameter: 14 mm), whose trajectories were recorded with a 10–15 Vicon camera system (Vicon-UK, Oxford, UK; sampling frequency of 100 Hz; built-in Woltring filter with mode MSE and smoothing of 15 mm^2^) during barefoot walking at self-selected speed on a 10-m walkway. Two embedded force plates (AMTI, Watertown, MA, USA; sampling frequency: 1500 Hz) captured ground reaction forces. Nexus software (Nexus 2.10. Vicon-UK, Oxford, UK) was used to define gait cycles and estimate trunk, pelvis and lower limb kinematic waveforms, lower limb kinetic waveforms, and spatiotemporal parameters. Ten gait cycles with kinematic data, of which three to five with kinetic data, were collected bilaterally. Quality of collected gait cycles was checked in a custom-made MATLAB software (The Mathworks Inc., Natick, M.A., 2016 and 2021b). We averaged the kinematic waveforms, kinetic waveforms and spatiotemporal parameters of the selected gait cycles with good quality per observation separately for the right and left sides. Each observation was classified into the mildly affected gait pattern, the tiptoeing gait pattern, or the flexion gait pattern according to our previously introduced gait classification [17]. Gait data of nineteen TD boys (Plug-In Gait Lower-body marker model; marker diameter: 14 mm) with a similar age range as the boys with DMD was selected from our reference database at the University Hospital Leuven. Data for the trunk segment was not collected in the TD boys, as only the lower-body marker model was applied.

**Fig. 1:**
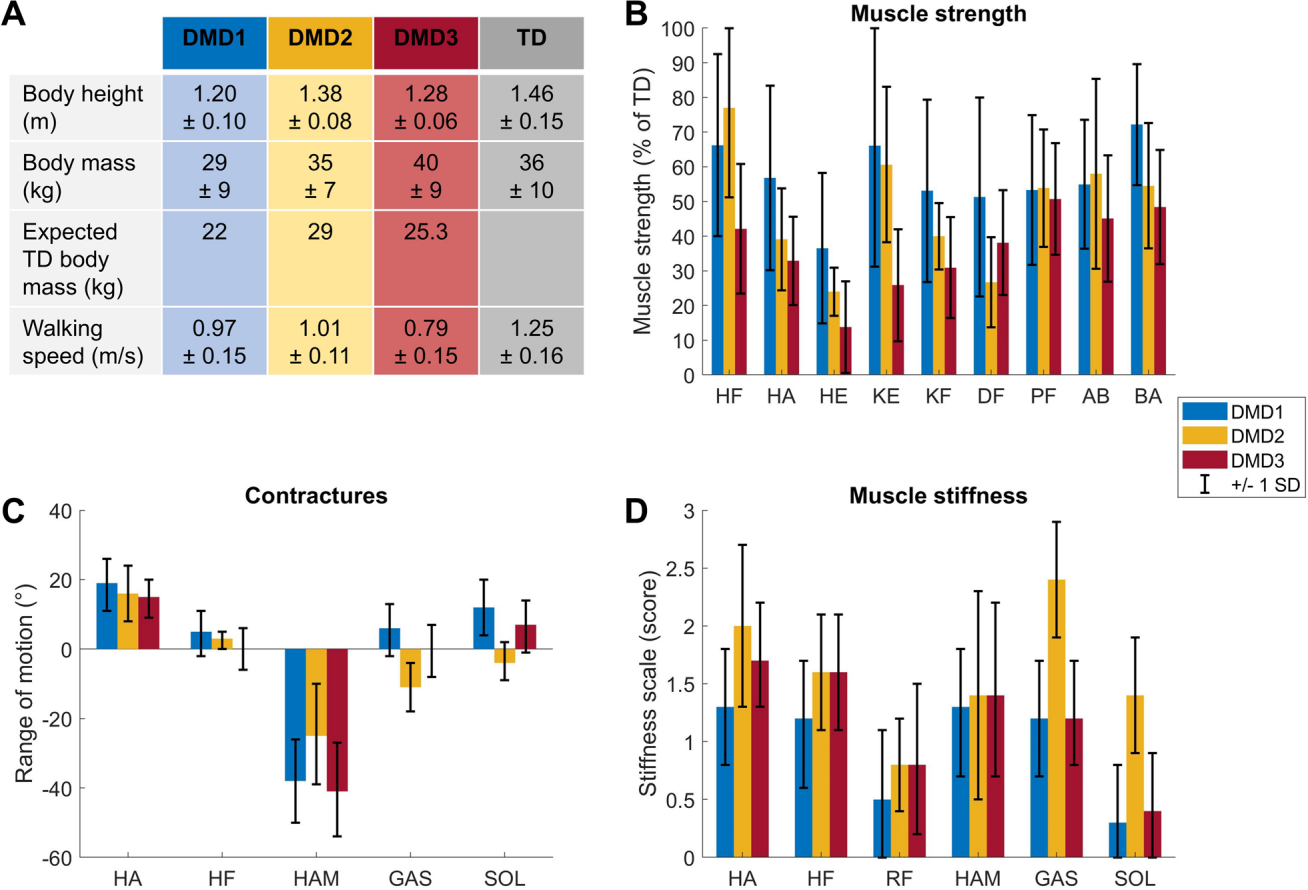
Experimental data used to personalize musculoskeletal models. **A** Anthropometry (mean ± 1 standard deviation). **B** Muscle strength deficits. **C** Passive range of motion. **D** Muscle stiffness. AB, abdominal; BA, back; DF, dorsiflexors; DMD1, DMD group with the mildly affected gait pattern; DMD2, DMD group with the tiptoeing gait pattern; DMD3, DMD group with the flexion gait pattern; GAS, gastrocnemius; HA, hip abductors; HAM, hamstrings; HE, hip extensors; HF, hip flexors; KE, knee extensors; KF, knee flexors; PF, plantar flexors; RF, rectus femoris; SOL, soleus; TD, typically developing

The entire database of strength assessments, clinical examinations, and 3D gait analyses was divided into three DMD groups based on the label of the gait classification [17]: “DMD1”, i.e., the mildly affected gait pattern, “DMD2”, i.e., tiptoeing gait pattern, and “DMD3”, i.e., flexion gait pattern (Fig. 1). The selected TD gait dataset was used to represent the TD group. For each group, the mean and standard deviation (SD) were calculated for the gait data. For the DMD groups, the mean and SD were also calculated for the strength deficits, the ROM deficits and the clinical stiffness scale. These values—mean, one SD above the mean and one SD below the mean—were later used as input to the musculoskeletal models.

### Personalizing musculoskeletal model

#### Generic musculoskeletal model

We used the musculoskeletal model proposed by D’Hondt et al. [23]. This model has 33 degrees of freedom (dofs) (pelvis-to-ground: 6 dofs, hip: 3 dofs, knee: 1 dof, ankle: 1 dof, subtalar: 1 dof, midtarsal: 1 dof, metatarsophalangeal: 1 dof, lumbar: 3 dofs, shoulder: 3 dofs, and elbow: 1 dof). 94 Hill-type muscle–tendon units (92 muscles according to OpenSim’s gait2392 model and the right and left intrinsic foot muscles) actuate the lower limb and lumbar joints. A right and left plantar fascia is included. Eight ideal torque actuators actuate the shoulder and elbow joints. Five Hunt-Crossley contact spheres per foot model the foot–ground contact. Passive joint torques with exponential stiffness and damping [38] were added to the lower limb and lumbar joints to represent the effects of unmodeled passive structures [20]. An adjustment we made compared to D’Hondt et al. [23] was fitting the exponential stiffness of the lumbar joint to experimental data of Panjabi et al. [39]. Muscle excitation-activation coupling was described by Raasch’s model [40, 41] and muscle–tendon interaction and the dependence of muscle force on fiber length and velocity by a Hill-type muscle model [42, 43]. Skeletal motion was modeled with Newtonian rigid body dynamics [44, 45].

#### Scaling of generic musculoskeletal model to child’s dimensions

For each DMD group and the TD group, the generic musculoskeletal model was linearly scaled to the mean anthropometry of that group, using the OpenSim scaling tool. For the TD group, the mean length and body mass of the TD group were therefore used. Since the boys with DMD have an increased body mass due to extra fat tissue, we first scaled the model parameters to the mean length of the DMD group and the expected mean body mass of TD children of the same length (i.e., expected TD body mass). The additional mass of the children with DMD, i.e. difference between the mean body mass of the DMD group and expected TD body mass was then divided with 2/3 over the trunk and pelvis segments, and with 1/3 over the lower limb segments. This decision was based on the observation that the additional mass was predominantly distributed around the abdomen and buttocks, with secondary accumulation in the legs due to increased intramuscular and subcutaneous fat. Notably, no excess mass was observed in the feet. Subsequently, we scaled the model parameters that are also dependent on anthropometry but were not scaled by the OpenSim scaling tool (e.g. muscles’ maximal isometric force) according to geometric similarity (Additional file 1).

#### Modeling impairments

The experimental data of the selected side was used as input for both the left and right sides of the musculoskeletal models to create symmetrical models. We chose this approach as muscle impairments typically manifest symmetrically in DMD [46].

The muscles in the model were represented as Hill-type muscle–tendon units (for a detailed description see [42, 43]). The muscle–tendon unit consists of an active contractile element in parallel with a passive element, which is in series with a tendon. The muscle force arises from both the active contractile component and the passive elastic element as:

1 $${F}_{m}={F}_{m}^{max}*\left({f}_{m}^{act}({\widetilde{l}}_{m} , {\widetilde{v}}_{m} ,a)+ {f}_{m}^{pass}({\widetilde{l}}_{m})\right)$$

where ${F}_{m}$ is muscle force, ${F}_{m}^{max}$ is maximal isometric force, ${f}_{m}^{act}$ is the active muscle force-length-velocity characteristic, ${\widetilde{l}}_{m}$ is normalized fiber length, ${\widetilde{v}}_{m}$ is normalized fiber velocity, $a$ is muscle activation, and ${f}_{m}^{pass}$ is the (non-linear) passive muscle force-length characteristic (see [43] for mathematical expression and visual representation of the characteristics).

The most common parametrization of this model [42] assumes that maximal isometric force (${F}_{m}^{max}$), and passive muscle (see Eq. (1)) and tendon stiffness are coupled. Therefore, they all scale with ${F}_{m}^{max}$. However, in DMD, active and passive muscle forces do not decrease simultaneously. The loss of contractile tissue is accompanied by its replacement with fat and fibrotic tissue, resulting in a decline in active muscle force while passive muscle stiffness increases. Therefore, we modeled muscle weakness by scaling only the active force component (${f}_{m}^{act}$), rather than scaling ${F}_{m}^{max}$ that also scales the passive elements:

2 $${f}_{m, DMD}^{act}={f}_{m, TD}^{act}* \left(\frac{{MVIC}_{DMD}}{{MVIC}_{TD}}\right)$$

where MVIC_DMD_/MVIC_TD_ is either the mean, one SD above the mean, or one SD below the mean of the experimentally measured strength deficit.

In DMD, contractile tissue is not only lost but also replaced by fat and fibrotic tissue, resulting in increased muscle stiffness and eventually leading to contractures. We modeled this by shifting the passive muscle force-length relationship to shorter fiber lengths through a reduction in the fiber length at which passive muscle force begins to develop. Neither slack length nor fascicle length was altered; only the position of the passive force-length curve was adjusted. We used the ROM measurements and clinical stiffness scale to estimate this shift. For the ROM measurements, we estimated the difference in fiber length at which the muscle starts to develop passive force between TD and DMD from the difference in joint angle at the end of ROM. The joint angle at the end of ROM of TD children was based on age-related reference data reported by Mudge et al. [33] and Sankar et al. [34]. To estimate the corresponding difference in fiber length, we multiplied the difference in measured joint angle at end ROM between TD and DMD (in radians) with the moment arm of the muscles in the anatomical position. This difference in fiber length was normalized to optimal fiber length to compute the shift of the passive force-length relationship. For the clinical stiffness scale, the normalized fiber length at which passive force starts to develop was assumed 1 when the clinical stiffness score was 0 (no increased resistance), 0.83 when the score was 1 (minimal increased resistance), 0.67 when the score was 2 (increased resistance), and 0.5 when the score was 3 (highly pronounced resistance) corresponding to a shift of respectively 0, 0.17, 0.33, and 0.5. We shifted the passive force-length relationship by the mean of the shifts estimated based on the ROM and clinical stiffness score.

Boys in DMD3 presented a midfoot break on the videos, a feature associated with reduced arch height and arch stiffness. To model this foot deformity, we reduced the height of all foot segments with 10% and decreased the strength of the intrinsic foot muscles with 50% [23], since activation of the intrinsic foot muscles stiffens the arch and DMD is primarily a muscle disorder.

### Predictive simulation of gait

Our previously described optimal control framework was used to simulate gait [20, 21]. We formulated simulations of gait as optimal control problems. Gait can then be simulated by solving for muscle controls that minimize a cost function, while imposing task constraints and the dynamics of a musculoskeletal model, without relying on experimental data. The task constraints for walking were the average forward speed of the pelvis and right-left symmetry. For each symmetrical model, we imposed the average experimentally measured gait speed. As contact between segments was not explicitly modeled, distance constraints were used to prevent segments to penetrate each other [20]. We scaled the imposed distances based on body height (Additional file 2).

We used a previously determined cost function, i.e. the integral of the weighted sum of squared metabolic energy rate $(\dot{E})$, muscle activations (*a*), joint accelerations (*u*_*a*_), and passive joint torques (*T*_*p*_):

3 $$J=\frac{1}{d}{\int }_{0}^{{t}_{f}}\left({w}_{1}*{\dot{E}}^{2}+{w}_{2}*{a}^{2}+{w}_{3}*{u}_{a}^{2}+ {w}_{4}*{T}_{p}^{2}\right)*dt$$

where *d* is the distance traveled, *t*_*f*_ is half gait cycle duration, *t* is time, and *w*_*1-4*_ are the weight factors. Weight factors w_1_ and w_4_ were scaled based on anthropometry (Additional file 2).

This cost function was selected based on the agreement between simulated and experimental gait patterns at self-selected speed in a healthy individual and evaluated in other conditions (range of gait speeds; weakness; prosthesis use) [20]. The model of Bhargava et al. [47] was used to calculate the metabolic energy of the muscles and was made continuously differentiable by approximating conditional statements with a hyperbolic tangent.

Our framework is implemented in MATLAB (The Mathworks Inc., USA). Skeletal dynamics is formulated using OpenSim (based on version 4.3), CasaDi [48] is used to formulate the optimal control problem and calculate derivatives, and IPOPT [49] to solve the optimal control problem. The code and documentation of the user friendly PredSim code that was used for this study can be found here [22]: https://github.com/KULeuvenNeuromechanics/PredSim. We used the gait data of a TD individual (available on github “IK_Bounds_Default.mot”) as the initial guess for all simulations.

### Analyses

Forward simulations were conducted based on a model that represented the mean weakness and contractures (± SD) in each group (and thus not for individual patients). For each DMD group, simulations were performed based on six models to study how weakness and contractures with different severities impact the walking pattern: (1) mean weakness; (2) weakness one SD below the mean; (3) weakness one SD above the mean; (4) mean weakness and mean contractures; (5) weakness and contractures one SD below the mean; and (6) weakness and contractures one SD above the mean. For DMD3, one additional simulation was performed, incorporating the midfoot break in addition to the mean weakness and mean contractures. For the TD group, a single predictive simulation on a model without impairments was conducted, serving as the reference. Contractures and foot deformities were not simulated in isolation as muscle weakness is the primary clinical symptom of DMD and the aim was to improve understanding of its interaction with contractures and foot deformities.

To validate whether the predictive simulations can capture DMD gait, we compared the experimental differences in gait kinematics and kinetics between DMD and TD with the differences in gait kinematics and kinetics simulated based on the models with impairments and the TD model. We focused on comparing experimental and simulated differences between TD and DMD kinematics and kinetics, as some of the differences between experimental and simulated gait patterns might be due to the use of different kinematic models.

To study the interaction between weakness and contractures, we compared the simulation based on the mean weakness model with the simulation based on the mean weakness and contractures model, for each DMD group. For DMD3, we also compared the simulation of both models to the simulation based on the mean weakness, contractures and midfoot break model to evaluate the additional effect of modeling foot deformities.

To evaluate the effect of progressive impairments, we compared the simulations based on the -1SD (i.e., one SD below the mean) and + 1SD (i.e., one SD above the mean) models to the simulations based on the mean models.

## Results

### Simulations based on models that reflect DMD-specific impairments capture DMD-specific gait impairments

**Fig. 2:**
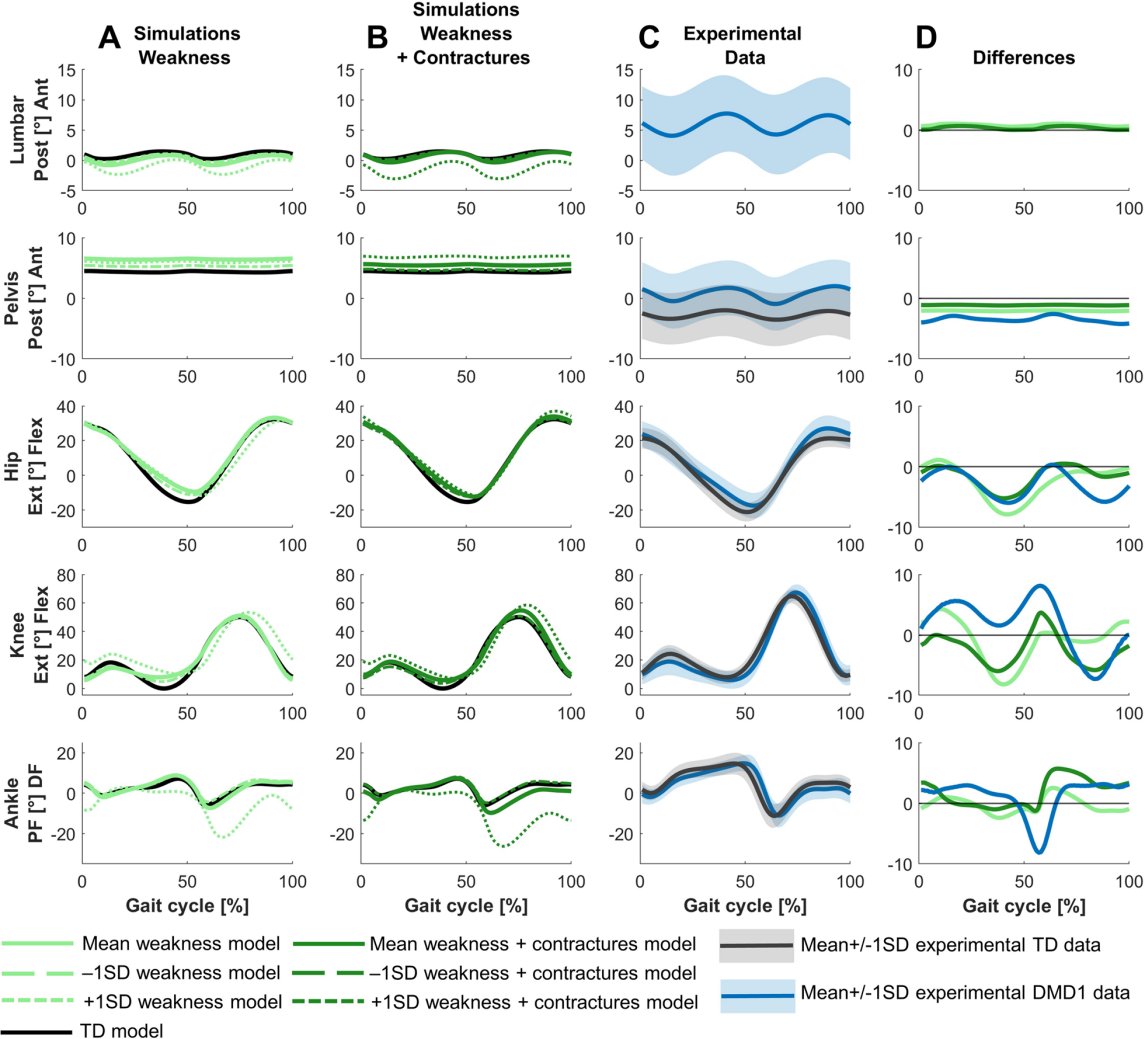
Comparison of simulated and experimental sagittal plane kinematics for DMD1. **A** Simulated kinematics when only modeling weakness. **B** Simulated kinematics when modeling weakness and contractures. **C** Experimental gait kinematics. **D** Differences in kinematics between the TD model and the mean weakness model (light green), the TD model and the mean weakness + contractures model (dark green), and the experimental TD and DMD1 data (blue). Ant, anterior; DF, dorsiflexion; Ext, extension; Flex, flexion; PF, plantar flexion; Post, posterior; TD, typically developing

**Fig. 3:**
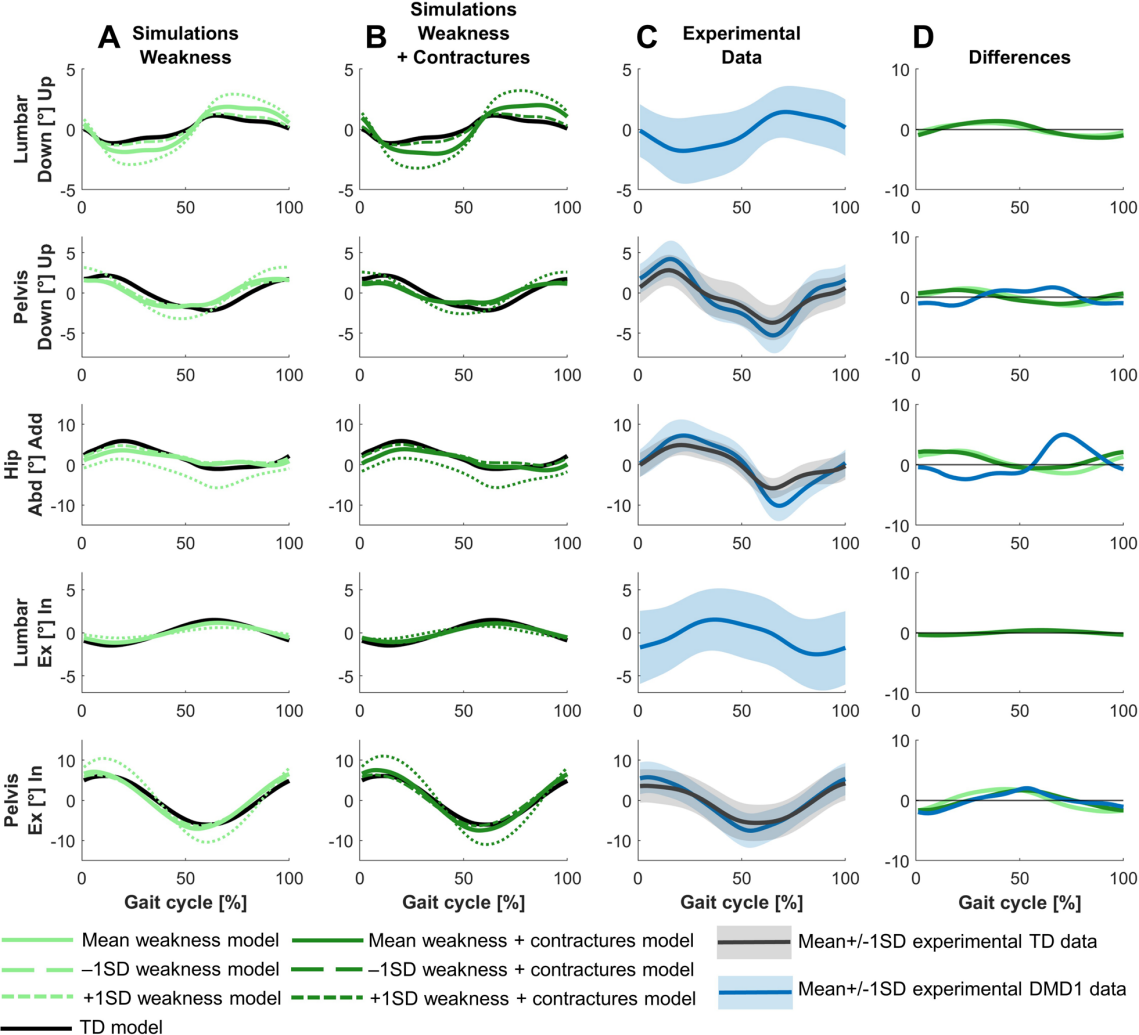
Comparison of simulated and experimental frontal and transverse plane kinematics for DMD1. **A** Simulated kinematics when only modeling weakness. **B** Simulated kinematics when modeling weakness and contractures. **C** Experimental gait kinematics. **D** Differences in kinematics between the TD model and the mean weakness model (light green), the TD model and the mean weakness + contractures model (dark green), and the experimental TD and DMD1 data (blue). Abd, abduction; Add, adduction; Ex, external; In, internal; TD, typically developing

**Fig. 4:**
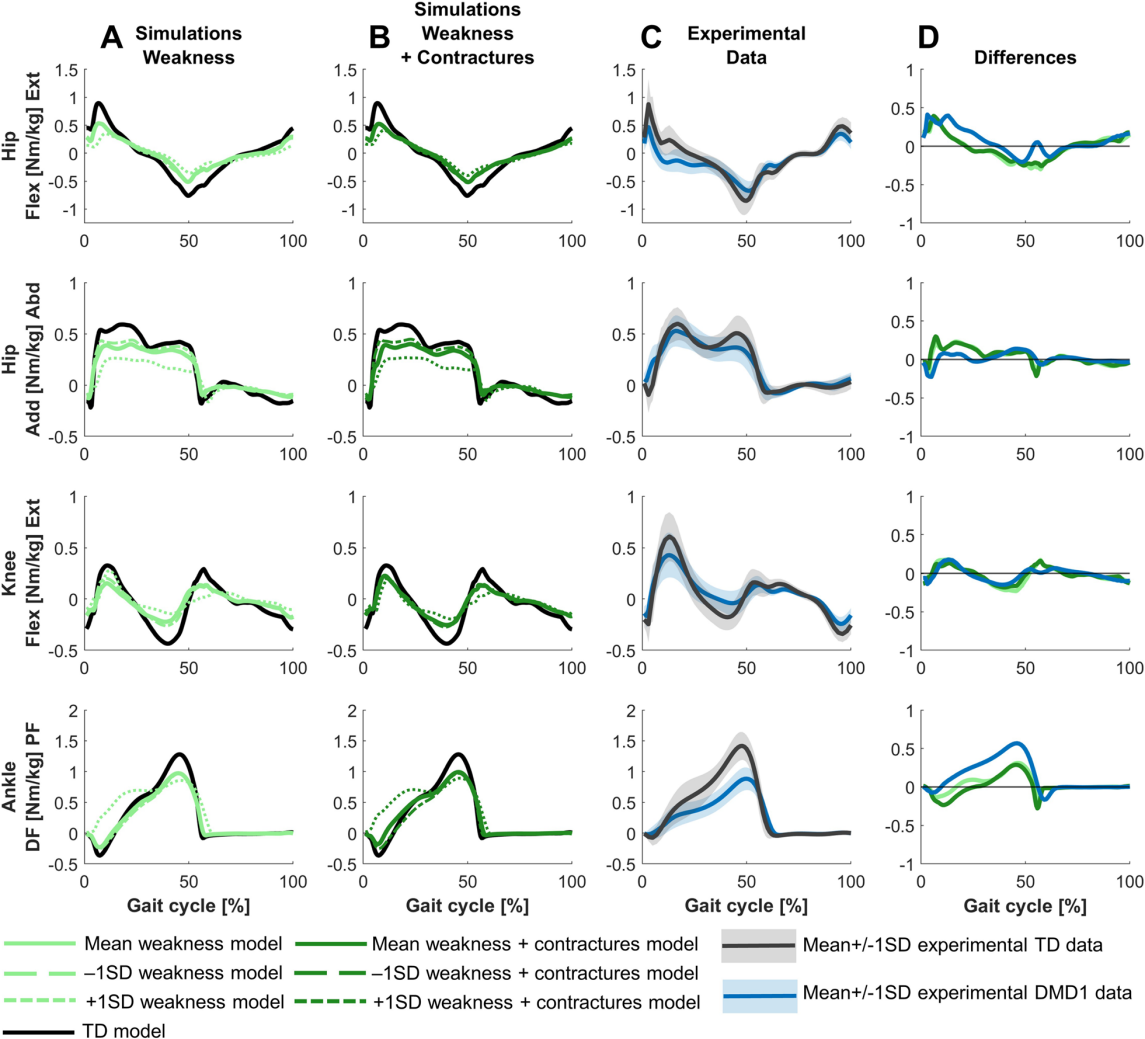
Comparison of simulated and experimental kinetics for DMD1. **A** Simulated kinetics when only modeling weakness. **B** Simulated kinetics when modeling weakness and contractures. **C** Experimental gait kinetics. **D** Differences in kinetics between the TD model and the mean weakness model (light green), the TD model and the mean weakness + contractures model (dark green), and the experimental TD and DMD1 data (blue). Abd, abduction; Add, adduction; DF, dorsiflexion; Ext, extension; Flex, flexion; PF, plantar flexion; TD, typically developing

For DMD1, the predictive simulation based on a model reflecting the mean weakness and contractures in this group captured the minor gait deviations between DMD1 and TD (Figs. 1–4). The experimental gait pattern of DMD1 deviated only minimally from TD gait. The main differences in the sagittal plane kinematics were increased anterior pelvic tilt, reduced hip extension at the end of stance, increased hip flexion during swing, reduced knee flexion motion during loading response, delayed knee flexion and increased peak knee flexion during swing, and reduced dorsiflexion during swing (Fig. 2). The increased anterior pelvic tilt and increased hip flexion during swing were minimally captured in the simulations. The reduced knee flexion motion during stance was captured, but the simulations predicted more flexion.

The main differences in the frontal and transverse plane kinematics were increased pelvic upwards position at initial contact, increased hip adduction during stance, and increased hip abduction during swing (Fig. 3). These differences were not fully captured in the simulations.

The main differences in joint moments were decreased hip extension moment, premature hip flexion moment (i.e., an early negative hip extension moment during stance), decreased knee extension and flexion moments, and reduced ankle plantar flexion moment (Fig. 4). The premature hip flexion moment was not captured in the simulations. The reduced ankle plantar flexion moment was captured, but the experimental difference was larger.

**Fig. 5:**
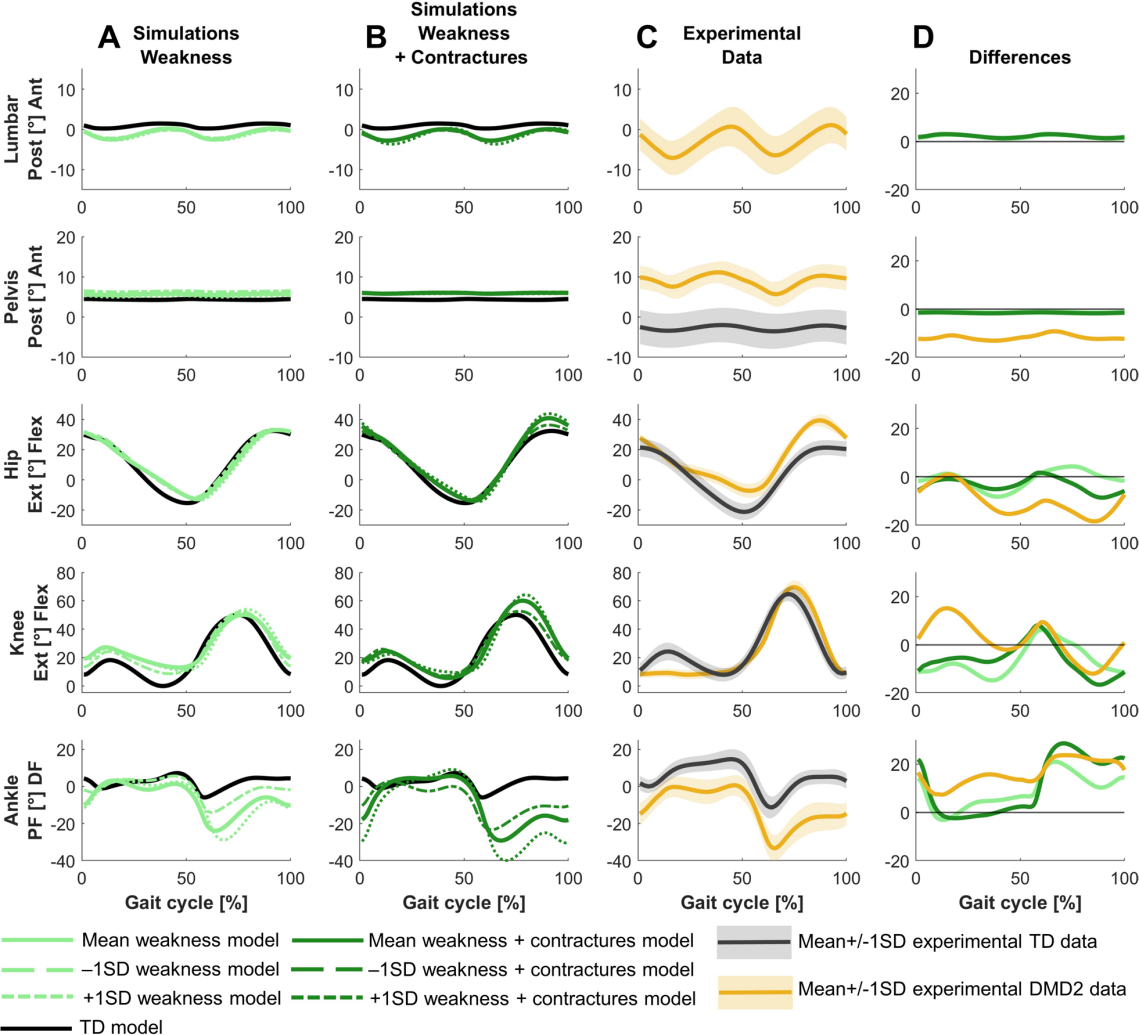
Comparison of simulated and experimental sagittal plane kinematics for DMD2. **A** Simulated kinematics when only modeling weakness. **B** Simulated kinematics when modeling weakness and contractures. **C** Experimental gait kinematics. **D** Differences in kinematics between the TD model and the mean weakness model (light green), the TD model and the mean weakness + contractures model (dark green), and the experimental TD and DMD2 data (yellow). Ant, anterior; DF, dorsiflexion; Ext, extension; Flex, flexion; PF, plantar flexion; Post, posterior; TD, typically developing

**Fig. 6:**
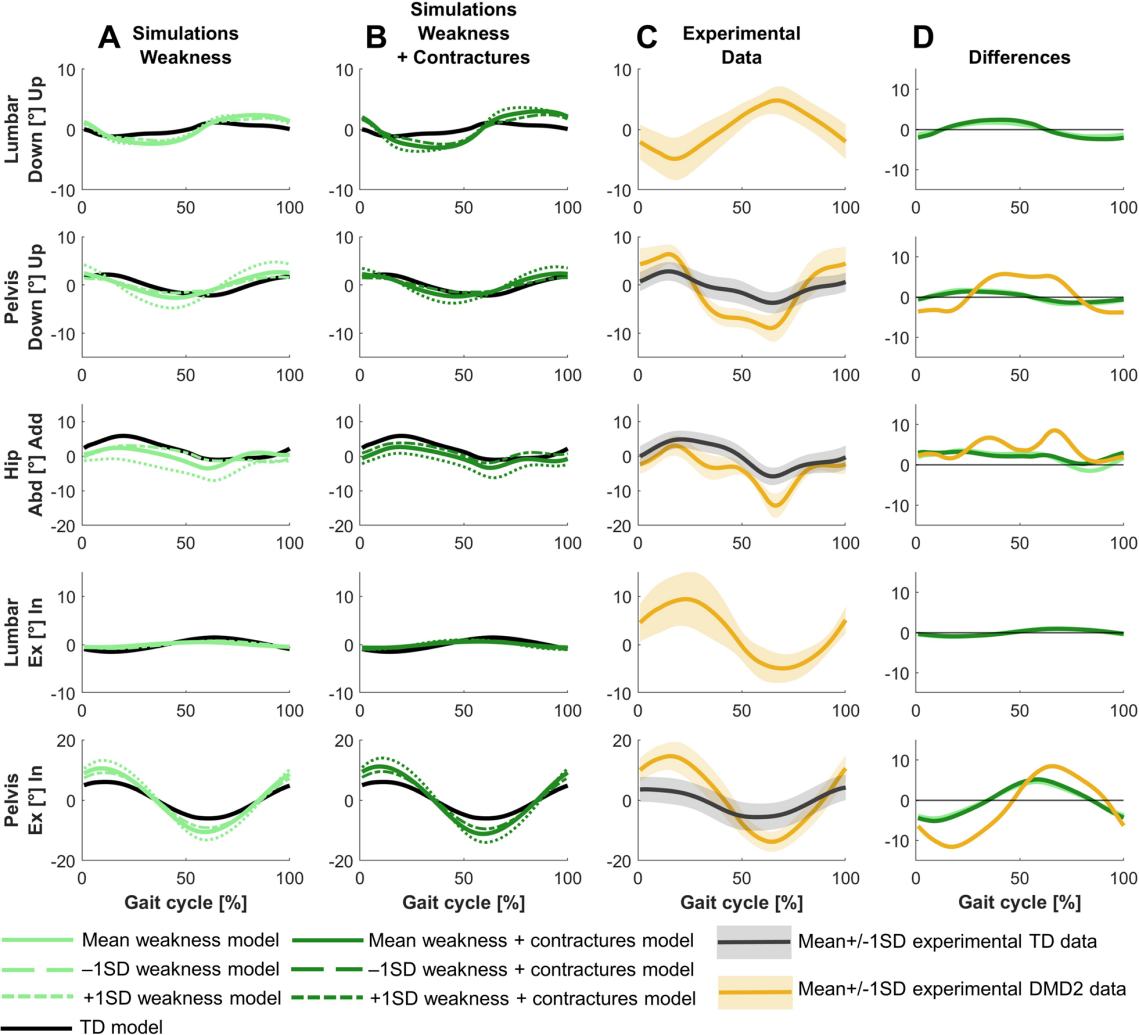
Comparison of simulated and experimental frontal and transverse plane kinematics for DMD2. **A** Simulated kinematics when only modeling weakness. **B** Simulated kinematics when modeling weakness and contractures. **C** Experimental gait kinematics. **D** Differences in kinematics between the TD model and the mean weakness model (light green), the TD model and the mean weakness + contractures model (dark green), and the experimental TD and DMD2 data (yellow). Abd, abduction; Add, adduction; Ex, external; In, internal; TD, typically developing

**Fig. 7:**
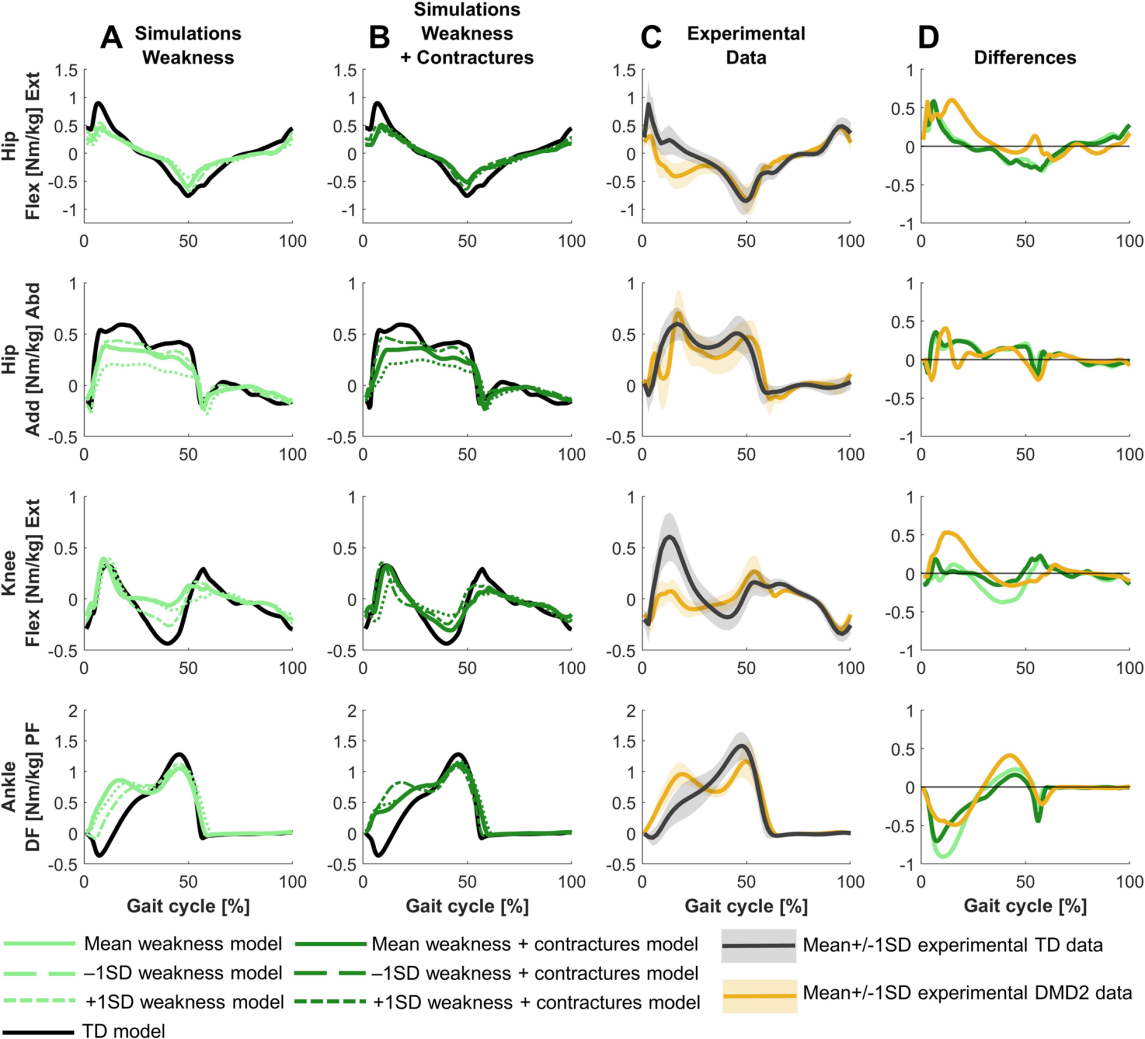
Comparison of simulated and experimental kinetics for DMD2. **A** Simulated kinetics when only modeling weakness. **B** Simulated kinetics when modeling weakness and contractures. **C** Experimental gait kinetics. **D** Differences in kinetics between the TD model and the mean weakness model (light green), the TD model and the mean weakness + contractures model (dark green), and the experimental TD and DMD2 data (yellow). Abd, abduction; Add, adduction; DF, dorsiflexion; Ext, extension; Flex, flexion; PF, plantar flexion; TD, typically developing.

For DMD2, the predictive simulation based on a model reflecting the mean weakness and contractures in this group captured many but not all deviations between DMD2 and TD (Fig. 1; Figs. 5–7). The experimental gait pattern of DMD2 was characterized by a tiptoeing pattern and deviated more from TD gait than DMD1. The main differences in the sagittal plane kinematics were continuous ankle plantar flexion, reversed first ankle rocker, horizontal second rocker, large increased plantar flexion during swing, absent knee flexion motion during loading response, delayed and increased peak knee flexion during swing, increased hip flexion at the end of stance and during swing, and increased anterior pelvic tilt (Fig. 5). The ankle pattern was captured in the simulations, yet the experimental difference in stance was larger. The absent knee flexion motion was not captured in the simulations. The increased hip flexion and anterior pelvic tilt were minimally captured in the simulations.

The main differences in the frontal and transverse plane kinematics were increased range of pelvic upwards/downwards and external/internal motion, and increased hip abduction in stance and swing (Fig. 6). The increased range of pelvic upwards/downwards motion was not fully captured in the simulations. The increased range of external/internal motion was captured, but the experimental difference was larger.

The main differences in joint moments were reduced hip extension moment, premature hip flexion moment, decreased knee extension and flexion moments, absent dorsiflexion moment, premature plantar flexion moment, and reduced peak plantar flexion moment (Fig. 7). Only the premature hip flexion moment and reduced knee extension moment were not captured in the simulations.

**Fig. 8:**
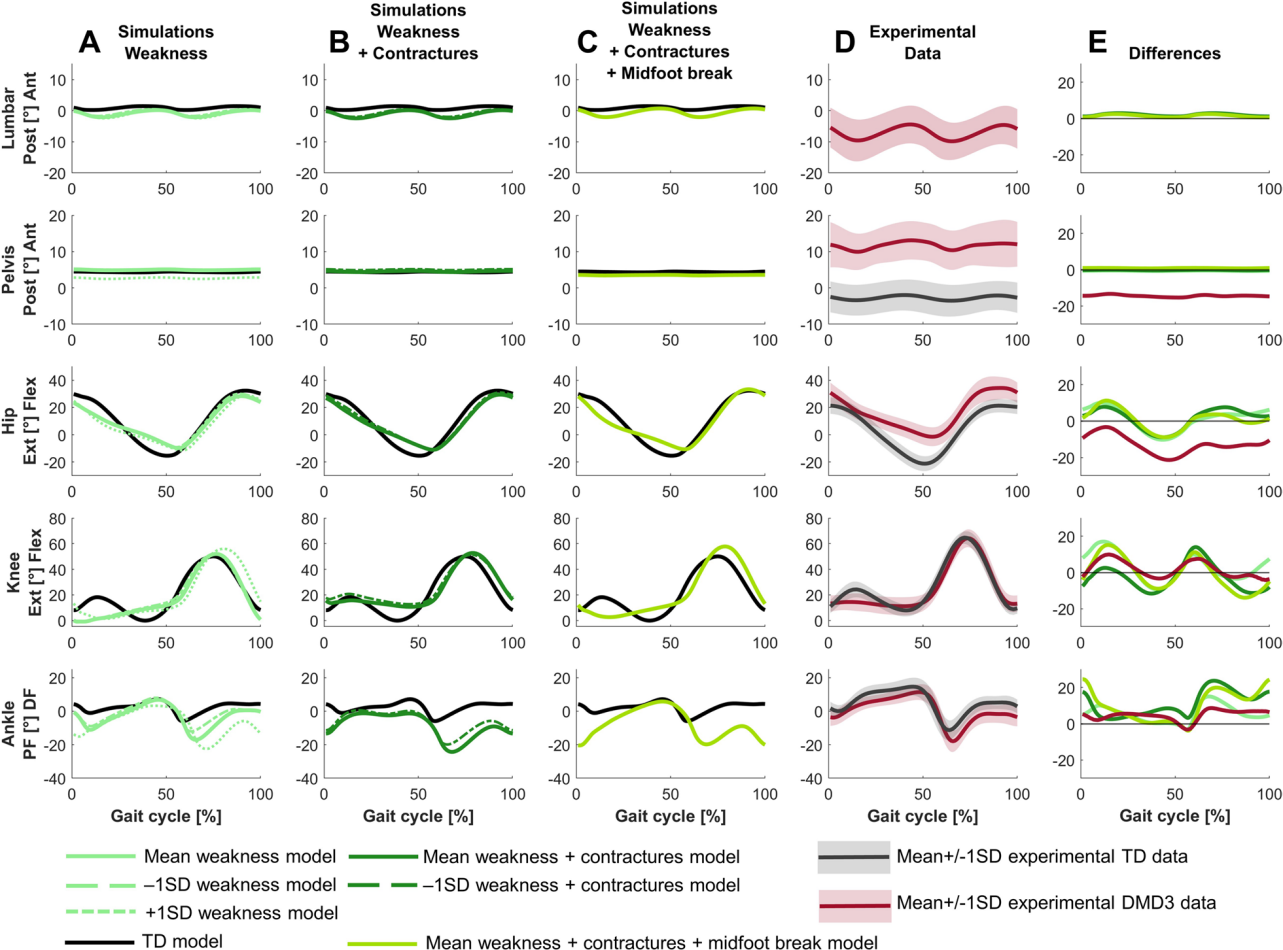
Comparison of simulated and experimental sagittal plane kinematics for DMD3. **A** Simulated kinematics when only modeling weakness. **B** Simulated kinematics when modeling weakness and contractures. **C** Simulated kinematics when modeling weakness, contractures and midfoot break. **D** Experimental gait kinematics. **E** Differences in kinematics between the TD model and the mean weakness model (light green), the TD model and the mean weakness + contractures model (dark green), the TD model and the mean weakness + contractures + midfoot break model (medium green), and the experimental TD and DMD3 data (red). Ant, anterior; DF, dorsiflexion; Ext, extension; Flex, flexion; PF, plantar flexion; Post, posterior; TD, typically developing

**Fig. 9:**
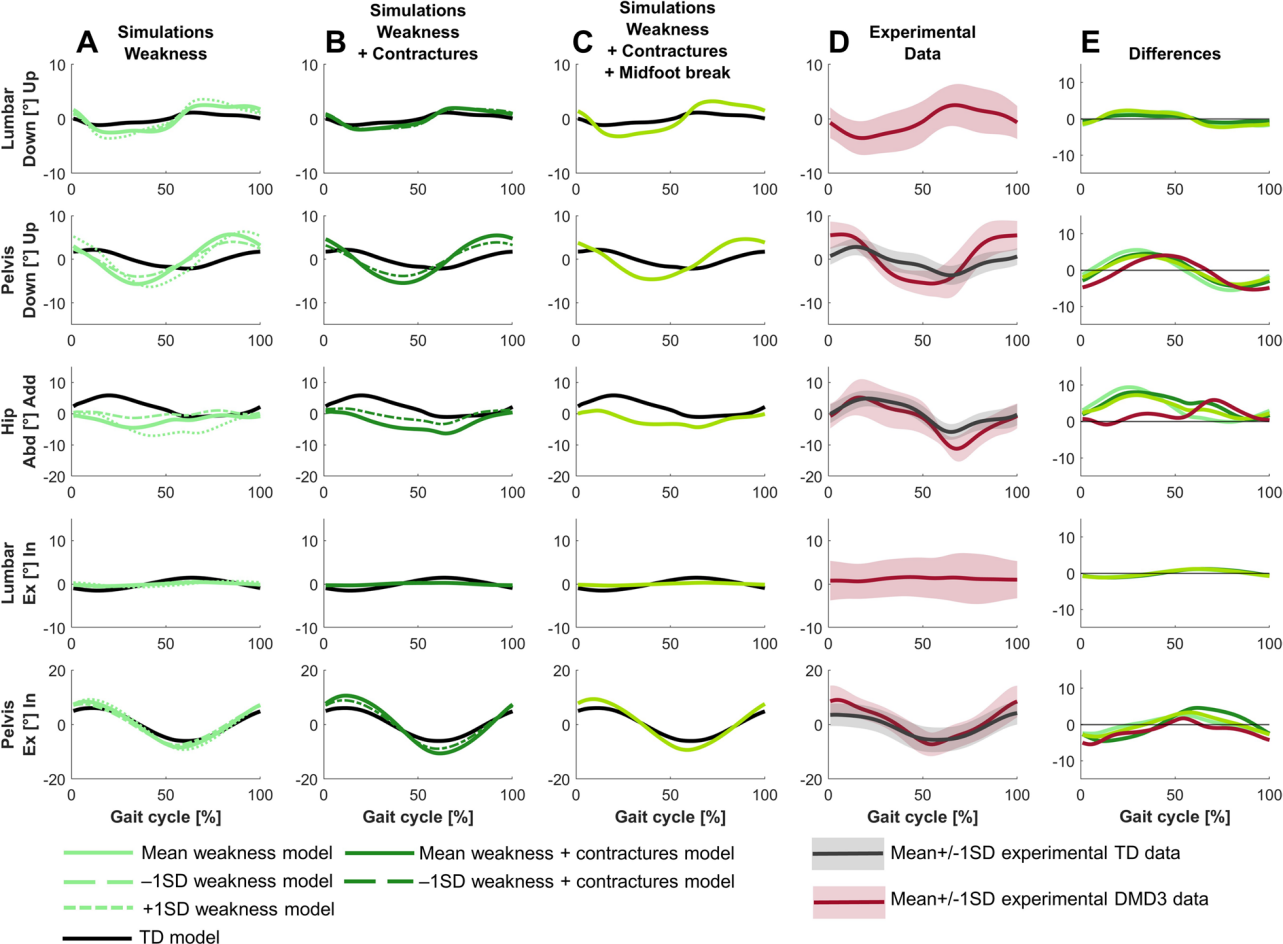
Comparison of simulated and experimental frontal and transverse plane kinematics for DMD3. **A** Simulated kinematics when only modeling weakness. **B** Simulated kinematics when modeling weakness and contractures. **C** Simulated kinematics when modeling weakness, contractures and midfoot break. **D** Experimental gait kinematics. **E** Differences in kinematics between the TD model and the mean weakness model (light green), the TD model and the mean weakness + contractures model (dark green), the TD model and the mean weakness + contractures + midfoot break model (medium green), and the experimental TD and DMD3 data (red). Abd, abduction; Add, adduction; Ex, external; In, internal; TD, typically developing

**Fig. 10:**
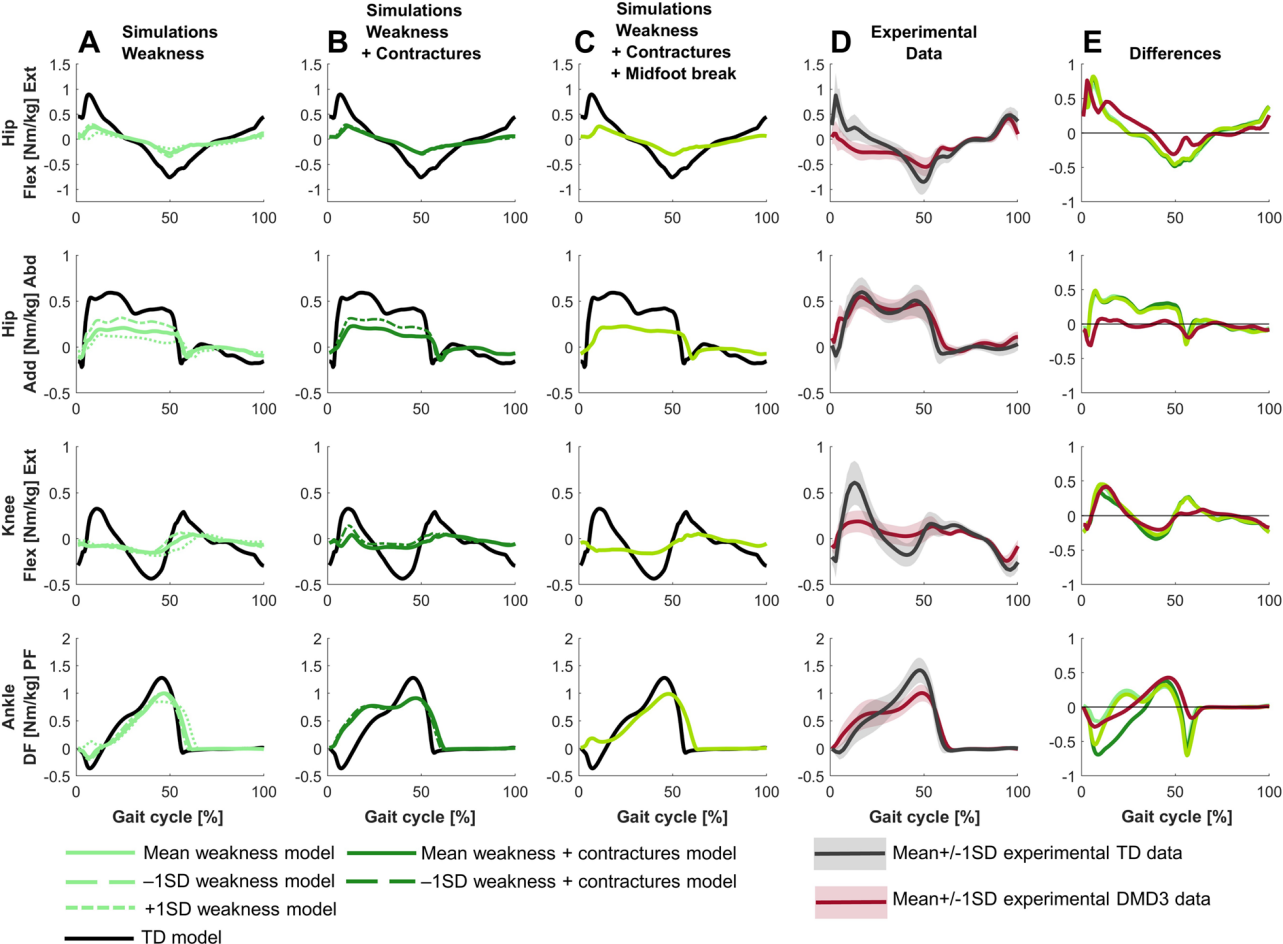
Comparison of simulated and experimental kinetics for DMD3. **A** Simulated kinetics when only modeling weakness. **B** Simulated kinetics when modeling weakness and contractures. **C** Simulated kinetics when modeling weakness, contractures and midfoot break. **D** Experimental gait kinetics. **E** Differences in kinetics between the TD model and the mean weakness model (light green), the TD model and the mean weakness + contractures model (dark green), the TD model and the mean weakness + contractures + midfoot break model (medium green), and the experimental TD and DMD3 data (red). Abd, abduction; Add, adduction; DF, dorsiflexion; Ext, extension; Flex, flexion; PF, plantar flexion; TD, typically developing

For DMD3, the predictive simulation based on a model reflecting the mean weakness, mean contractures and midfoot break in this group captured most but not all deviations between DMD3 and TD (Fig. 1, 8, 9, 10). The experimental gait pattern of DMD3 was characterized by a flexion pattern with distinct anterior pelvic tilt and posterior trunk leaning and deviated more from TD gait than DMD1. The main differences in the sagittal plane kinematics were increased anterior pelvic tilt, continuous increased hip flexion, delayed peak knee flexion during swing, absent knee flexion motion during loading response but increased knee flexion compared to DMD2, reduced dorsiflexion during swing and at initial contact (Fig. 8). The increased anterior pelvic tilt was not captured and the increased hip flexion was only minimally captured, mainly at the end of stance in the simulations. The delayed knee flexion during swing was captured, but simulations predicted also increased peak knee flexion. The reduced dorsiflexion during swing and at initial contact was captured, but simulations predicted more plantar flexion.

The main differences in the frontal and transverse plane kinematics were increased range of pelvic upwards/downwards and external/internal motion, but less pronounced than in DMD2, and increased hip abduction in swing (Fig. 9). The simulations predicted more hip abduction during stance than experimentally observed.

The main differences in joint moments were absent hip extension moment, premature hip flexion moment, reduced peak hip flexion moment, decreased knee extension and flexion moments, absent dorsiflexion moment, premature plantar flexion moment, and reduced peak plantar flexion moment (Fig. 10). The premature hip flexion moment was not captured in the simulations and the simulation predicted a decrease in hip abduction moment that was not observed experimentally.

### Contractures and foot deformities contribute to gait impairments in DMD

For DMD1, mainly weakness captured the differences between the mildly affected gait pattern and TD gait (Figs. 2–4). Yet, by adding the contractures to the weakness, additional gait deviations were explained such as reduced dorsiflexion in swing and at initial contact, and the delayed knee flexion and increased peak knee flexion during swing.

For DMD2, weakness already captured most differences between the tiptoeing gait pattern and TD gait (Figs. 5–7). The pattern at the ankle was effectively reflected by only modeling muscle weakness. However, additional gait deviations were captured by modeling contractures in addition to weakness, such as increased hip flexion, knee flexion, and plantar flexion during swing. The loss of knee flexion motion during loading response was not adequately captured by either muscle weakness or the combination of weakness and contractures.

For DMD3, weakness already explained the majority of the differences between the flexion gait pattern and TD gait (Figs. 8–10). In contrast to DMD2, the loss of knee flexion motion during loading response was effectively captured by muscle weakness in DMD3. Some additional gait differences were reflected by adding contractures to weakness, such as increased hip abduction during swing. However, a tiptoeing pattern (i.e., the main characteristic of DMD2) at the ankle with increased knee flexion was predicted by combining weakness and contractures, though this was not observed experimentally. By modeling a midfoot break in addition to the weakness and contractures, this pattern shifted towards increased dorsiflexion at the end of stance and reduced knee flexion during loading response, which better reflected experimental observations.

### The effect of progressive impairments

For DMD1, simulations based on the less affected −1SD models, reflecting muscle impairments one SD below the mean, closely resembled simulations based on the mean models, both when modeling weakness in isolation and weakness and contractures (Figs. 2–4). In contrast, simulations based on the more affected +1SD models, reflecting muscle impairments one SD above the mean, clearly differed from simulations based on the mean models and exhibited some gait features that were observed in more severely affected children with DMD, such as tiptoeing at the ankle.

For DMD2, simulations based on −1SD and +1SD models differed from the simulations of the mean models (Figs. 5–7). Simulations based on the −1SD models had less severe deviations, while simulations based on +1SD models had more severe deviations. The tiptoeing gait pattern at the ankle was consistently present across all simulations based on these ±1SD models, both when modeling weakness in isolation, and weakness and contractures.

For DMD3, simulations based on the less affected −1SD models closely resembled simulations based on the mean models (Figs. 8–10). The simulations based on the more affected +1SD weakness model differed slightly more from simulations based on the mean models, with more severe gait deviations such as increased drop foot during swing, increased plantar flexion at initial contact with a reversed first ankle rocker, increased pelvic downwards/upwards range of motion, and increased hip abduction during swing. Interestingly, simulations based on the more affected +1SD weakness and contractures model could not find a feasible solution.

## Discussion

Most but not all key features of the three DMD gait patterns were captured in the predictive simulations based on models reflecting DMD-specific muscle weakness and contractures. Overall, the simulated differences between TD and DMD gait patterns more closely matched the experimental differences when stiffness/contractures were modeled alongside muscle weakness. This suggests that not only muscle weakness but also muscle stiffness/contractures contribute to the pathological gait pattern. Interestingly, the tiptoeing gait pattern (DMD2) was observed when only modeling weakness, suggesting that weakness causes tiptoeing even in the absence of contractures. For DMD3, the experimental differences were even better captured when foot deformities (midfoot break) were included in addition to weakness and stiffness/contractures. Our current results highlight the potential of predictive simulations to study the effect of progressive weakness and contractures. Not only could the predictive simulations capture a mildly affected gait pattern and two more severely affected gait patterns based on the severity and location of the muscle impairments, but they also predicted less affected gait features for less severe impairments (−1SD) and more affected gait features (DMD1 and DMD2) or even the inability to walk (DMD3) for more severe impairments (+1SD).

Despite the assumptions and the limited accuracy of the clinical measurements, the models and predictive simulations were able to capture the main features of DMD gait. This suggests that models can be personalized on a population-based level using clinical measurements. This is especially promising given that clinical measurements are sparse (e.g. no assessment of individual muscles), and depend on the cooperation of the boys with DMD and/or the interpretation of the clinician. The instrumented strength assessment is proven reliable [31] but depends on the child’s cooperation and might therefore not reflect the maximal force [50]. The clinical examination, including the range of motion measurements and the clinical stiffness scale, is subjective and depends on the assessor. Additionally, these clinical measurements were expressed as deficits relative to TD children, which could introduce an additional error if these measurements in TD children were also not performed accurately. Personalizing the active muscle force based on the experimentally measured strength outcomes is relatively straightforward. However, several assumptions were made when modeling contractures. In particular, it is unclear how clinical stiffness scores relate to alterations in the muscle’s passive force-length behavior. In a Hill type model, passive muscle force is a non-linear function of muscle length. Here, we chose to model contractures as a shift of the passive force-length relationship, i.e., a decrease in muscle length at which passive force starts to develop. This captures the increased resistance at smaller muscle stretches but it is possible that the tissue stiffness also increases. Future studies should experimentally measure passive torque-angle relationships and use this information to validate and if needed adapt the proposed modeling workflow. For accurate predictive simulations, it is sufficient that we capture the passive torque across postures. This can be obtained with a Hill model but a better understanding of how passive tissue properties change is needed to inform personalization of the passive force-length relationship. On the other hand, more complex muscle models [54–56] could give valuable insights into the relationship between changes in muscle microstructure and alterations in the muscle’s overall mechanical behavior [55, 56], and when informed with experimental data on muscle microstructure could outperform Hill-type muscle models.

To further evaluate the robustness of our findings against modeling errors, we evaluated the sensitivity of the differences between simulated TD and DMD kinematics and kinetics to the amount of muscle weakness and contractures separately (Additional files 3–5). The simulations in which weakness and contractures were simultaneously altered by 1SD already give an indication of the sensitivity of our simulations to model parameters. Additionally, we performed three further analyses. In the first, muscle weakness was held constant at its mean value, while contractures were progressively increased by shifting the passive force-length curve to 0.9, 0.8, and 0.7 of the normalized fiber length (Additional file 3). The results indicate that shifting the passive curve by 0.1 had only a small effect on the differences between simulated TD and DMD kinematics and kinetics. Given the non-linear relationship between model parameters and gait kinematics and kinetics, we varied contractures around their mean value by ±1 SD while holding muscle weakness constant at its mean value (see Additional file 4). The results showed minimal effects of varying the shift of the passive force-length curve by 0.04 to 0.1 on the differences between simulated TD and DMD kinematics and kinetics. Together, these analyses suggest that small deviations (i.e., a shift about 0.1) in contracture estimation do not substantially affect the outcomes. Only increasing contractures by +1SD for DMD1 had a larger impact and resulted in larger differences between the simulated and experimental differences as the simulated gait pattern began to resemble the gait pattern of DMD2. This suggests that the proposed estimates of the shift are more realistic than larger shifts introducing larger contractures. Finally, in the third analysis, we varied muscle weakness around its mean value by ±1SD while holding contractures constant at their mean value (Additional file 5). Here, the impact of altering muscle strength by 7% to 35% was greater than that of varying the shift of the passive force-length curve by 0.04 to 0.1 (Additional file 4), indicating that the simulations are more sensitive to muscle weakness than contractures. However, we also believe that our muscle weakness estimates are more accurate due to the use of quantitative data.

The proposed approach can also be used to create personalized models for individual patients. However, further validation is required as measurement errors (assessments of muscle weakness and contractures) might be larger for individual patients than on a population level where we could use a mean value across many patients. Whereas asymmetry is typically limited, it has been observed in some patients. Patient-specific simulations could further elucidate the effect of asymmetric weakness or contractures on the gait pattern.

Simulations based on a cost function that was designed for simulations of unimpaired gait captured key features of DMD gait, suggesting that motor control is largely unaffected by DMD. The cost function used here was selected because it best captured unimpaired gait at self-selected speed [20]. Our simulation results confirm the underlying assumption that motor control of children with DMD is unimpaired. However, it is possible that we need to consider additional factors in the cost function, such as avoiding eccentric contractions or pain, to predict fine details of DMD gait. For example, in DMD2, we were unable to replicate the loss of knee flexion motion during loading response because this group still had sufficient strength in the knee extensors, unlike DMD3. Therefore, it is possible that children with DMD adopt this pattern to avoid eccentric contraction or pain [57] in the knee extensors, despite having sufficient strength. Further consideration of these factors in the cost function may enhance the accuracy and applicability of the simulations.

The difference in mass and mass distribution between the TD group and DMD groups could have influenced the simulated gait patterns [21]. We aimed to reflect the altered anthropometry of boys with DMD by distributing the additional mass compared to TD with 2/3 over the trunk and pelvis segments (reflecting that additional fat tissue is predominantly distributed around the abdomen and buttocks) and with 1/3 over the lower limb segments (reflecting secondary fat accumulation due to increased intramuscular and subcutaneous fat in the legs). The mass and mass distribution were therefore not constant across the different models. Falisse et al. [21] investigated the influence of mass distribution on simulations and found that a lighter torso and heavier legs produced slightly larger knee flexion angles, knee extension moments, and knee extensor activations during stance. Consequently, the different mass and mass distribution of the models could have influenced the differences between simulated gait patterns. We adjusted only the mass and inertia. However, the accumulation of fat tissue around the abdomen shifts the trunk’s center of mass forward. Not accounting for this shift in the center of mass may have contributed to the predictive simulations’ limited ability to capture the increase in posterior trunk leaning and premature hip flexion moment. Future studies should investigate the impact of mass, mass distribution, and trunk center of mass on the simulated gait patterns.

Muscle weakness but also contractures contribute to DMD gait. Previously, muscle weakness was considered the most important factor influencing pathological gait in DMD [1, 5], whereas the effect of contractures, in addition to muscle weakness, was poorly understood. Our predictive simulations indicate that muscle weakness is the main contributor to DMD gait deviations but some gait features were only predicted by modeling the interaction between muscle weakness and contractures. Therefore, the role of contractures cannot be underestimated, as they also contribute to the pathological gait patterns.

Altered foot properties might have an important contribution to the flexion gait pattern (DMD3). When the midfoot break was not modeled, a tiptoeing gait pattern was predicted, which did not match the experimental data. Videos of some boys with DMD showed a premature heel rise with abnormally increased motion through the midfoot (i.e., a midfoot break), leading to a dorsiflexion overestimation in the experimental data due to the applied simplistic marker foot model. The midfoot break might be caused by more flexible feet with larger arch drops and lower arch rigidity, secondary from obesity, combined with the plantar flexion contracture restricting the ankle dorsiflexion [17]. When a midfoot break was included in the model, there was still tiptoeing gait at initial contact, but dorsiflexion increased again at terminal stance, and knee flexion decreased during loading response, better corresponding to the experimental gait. This highlights the importance of using detailed, complex 3D foot models and modeling foot deformities to accurately capture pathological gait patterns.

Predictive simulations have the potential to study the effect of progressive impairments on the DMD gait pathology. Our exploration based on models that were less or more impaired yielded plausible results and led to testable hypotheses. For example, when increasing weakness but not stiffness and weakness in DMD3 a gait pattern could still be found thereby suggesting that increases in stiffness are more threatening for gait function than increases in weakness. Future experimental research should test if increases in stiffness indeed play a crucial role in the loss of ambulation. Furthermore, future work is needed to examine the individual effects of the progressive weakness and contractures in different muscle groups to gain insights into potential supportive interventions, such as the use of exoskeletons.

The differences between experimental and simulated gait patterns arise from two factors: the use of different models and the inaccuracies of the predictive simulations. As demonstrated in a previous study [58], the Plug-in Gait and OpenSim models are not fully comparable with kinematic differences up to 13°. The majority of these discrepancies are attributed to differences in the anatomical models, including different anatomical segment frames and joint constraints, rather than differences in computational methods (direct versus inverse kinematics). The largest difference is observed in pelvis anterior/posterior tilt angles. This can be attributed to different definitions of neutral pelvis tilt. In the OpenSim model neutral pelvis tilt is aligned with the anatomical position, whereas in the Plug-in Gait model neutral pelvis tilt is based on the pelvic markers, resulting in a 13° offset between the two reference systems. We did account for this offset between Nexus and OpenSim, but our marker set did not allow us to perform inverse kinematics in OpenSim for the experimental data based on the model we used for simulations. In particular, we did not have enough markers on the feet to estimate detailed foot and ankle kinematics. The use of two different models primarily affects kinematics. We, therefore, focused on comparing the differences between DMD and TD kinematics but this comparison may still be slightly influenced by the use of different kinematic models. However, not all discrepancies between experimental and simulated gait can be attributed to differences in modeling joint kinematics. Predictive simulations do not fully capture experimental kinematics and kinetics. For example, the simulations underestimate peak knee flexion during swing. This might be due to errors in modeling quadriceps properties in the generic model leading to unrealistically large passive forces when the knee is deeply flexed. Additionally, the simulations failed to capture the large increases in anterior pelvic tilt. We used an existing model of musculoskeletal geometry that is most frequently used (OpenSim’s gait2392 model [51]). The trunk model is simplified with only one joint, and the muscle anatomy at the hip/pelvis is not very detailed. For instance, the psoas muscle in the musculoskeletal model [51] inserts at the pelvis instead of the lumbar vertebrae. This might explain the underestimated increase in anterior pelvic tilt and posterior trunk leaning, and the absent premature hip flexion moment in the simulations. Furthermore, the segment lengths of the generic adult model were scaled to those of children but the differences in bone shapes were not yet considered. For example, the femoral anteversion angle decreases from 40° to 15°, and the neck-shaft angle from 140° to 125°, from birth to skeletal maturity [52, 53], which affects the moment arms of the muscles. Nevertheless, this model provides the most realistic predictive simulations of gait [25] and we deemed it sufficiently realistic to study the effect of weakness and contractures on the gait pattern. However, refining the anatomical details and incorporating more accurate passive force-length relationship measurements might enhance the predictive accuracy of these simulations.

## Conclusion

Predictive simulations based on personalized models capture key features of DMD gait patterns. While muscle weakness was primarily responsible for gait deviations, muscle stiffness/contractures and foot deformities further contributed to gait deviations. These results highlight the potential of predictive simulations to improve our understanding of the causal relationships between progressive impairments and pathological gait patterns in boys with DMD. Increased understanding of the differential effect of weakness and stiffness might guide treatment selection.

## Supplementary Information

Additional file 1.

Additional file 2.

Additional file 3.

Additional file 4.

Additional file 5.

## Data Availability

All data concerning this study is available within the manuscript. Detailed data is available upon reasonable request to the first author.

## Abbreviations

*a*: Muscle activations
Dofs: Degrees of freedom
DMD: Duchenne muscular dystrophy
$\dot{E}$: Metabolic energy rate
${f}_{m}^{act}$: Active force component of the force-length relationship
${F}_{m}^{max}$: Maximal isometric force
MVIC: Maximal voluntary isometric contractions
ROM: Range of motion
SD: Standard deviation
TD: Typically developing
*T*_*p*_: Passive joint torques
*u*_*a*_: Joint accelerations

## References

[1] Sussman M. Duchenne muscular dystrophy. J Am Acad Orthop Surg. 2002;10:138–51. 10.5435/00124635-200203000-00009. 11929208 10.5435/00124635-200203000-00009

[2] Jones D, Round J, de Haan A. Skeletal muscles, from molecules to movement. A textbook of muscle physiology for sport exercise, physiotherapy and medicine. London, UK: Elsevier; 2010.

[3] Deenen JCW, Horlings CGC, Verschuuren JJGM, Verbeek ALM, Van Engelen BGM. The epidemiology of neuromuscular disorders: a comprehensive overview of the literature. J Neuromuscul Dis. 2015;2:73–85. 28198707

[4] Goemans N, Signorovitch J, McDonald C, Mercuri E, Niks E, Wong B, et al. Functional trajectories of upper limb and pulmonary function before and after loss of ambulation in Duchenne muscular dystrophy. Neuromuscul Disord. 2021;31:S47-162. 10.1016/j.nmd.2021.07.148.

[5] Sutherland DH, Olshen R, Cooper L, Wyatt M, Leach J, Mubarak S, et al. The pathomechanics of gait in Duchenne muscular dystrophy. Dev Med Child Neurol. 1981;23:3–22. 10.1111/j.1469-8749.1981.tb08442.x. 7202868 10.1111/j.1469-8749.1981.tb08442.x

[6] Rideau Y, Duport G, Delaubrier A, Guillou C, Renardel-Irani A, Bach J. Early treatment to preserve quality of locomotion for children with Duchenne muscular dystrophy. Semin Neurol. 1995;15:9–17. 10.1055/s-2008-1041001. 7638464 10.1055/s-2008-1041001

[7] Bakker JP, de Groot IJ, Beckerman H, de Jong BA, Lankhorst GJ. The effects of knee-ankle-foot orthoses in the treatment of Duchenne muscular dystrophy: review of the literature. Clin Rehabil. 2000;14:343–59. 10.1191/0269215500cr319oa. 10945419 10.1191/0269215500cr319oa

[8] Khodadadeh S, McClelland MR, Patrick JH, Edwards RH, Evans GA. Knee moments in Duchenne muscular dystrophy. Lancet. 1986;2:544–5. 10.1016/s0140-6736(86)90114-5. 2875283 10.1016/s0140-6736(86)90114-5

[9] de Souza MA, Figueiredo MM, de Baptista CR, Aldaves RD, Mattiello-Sverzut AC. Beneficial effects of ankle-foot orthosis daytime use on the gait of Duchenne muscular dystrophy patients. Clin Biomech. 2016;35:102–10. 10.1016/j.clinbiomech.2016.04.005. 10.1016/j.clinbiomech.2016.04.005 27139255

[10] Eagle M. Report on the Muscular dystrophy campaign workshop: exercise in neuromuscular diseases Newcastle, January 2002. Neuromuscul Disord. 2002;12:975–83. 10.1016/s0960-8966(02)00136-0. 12467755 10.1016/s0960-8966(02)00136-0

[11] Markati T, Oskoui M, Farrar MA, Duong T, Goemans N, Servais L. Emerging therapies for Duchenne muscular dystrophy. Lancet Neurol. 2022;21:814–29. 10.1016/S1474-4422(22)00125-9. 35850122 10.1016/S1474-4422(22)00125-9

[12] Ricci G, Bello L, Torri F, Schirinzi E, Pegoraro E, Siciliano G. Therapeutic opportunities and clinical outcome measures in Duchenne muscular dystrophy. Neurol Sci. 2022;43:625–33. 10.1007/s10072-022-06085-w. 35608735 10.1007/s10072-022-06085-w PMC9126754

[13] Goemans N, Van den Hauwe M, Signorovitch J, Swallow E, Song J, Colloborative Trajectory Analysis Project (cTAP). Individualized prediction of changes in 6-minute walk distance for patients with Duchenne muscular dystrophy. PLoS ONE. 2016;11: e0164684. 10.1371/journal.pone.0164684. 27737016 10.1371/journal.pone.0164684 PMC5063281

[14] Goemans N. Therapy development and clinical outcome measures for Duchenne muscular dystrophy (PhD thesis). Leuven: KU Leuven; 2013.

[15] Gaudreault N, Gravel D, Nadeau S. Evaluation of plantar flexion contracture contribution during the gait of children with Duchenne muscular dystrophy. J Electromyogr Kinesiol. 2009;19:180–6. 10.1016/j.jelekin.2007.09.004. 10.1016/j.jelekin.2007.09.004 17977021

[16] Vandekerckhove I, Molenberghs G, Van den Hauwe M, Goemans N, De Waele L, Van Campenhout A, et al. Longitudinal interaction between muscle impairments and gait pathology in growing children with Duchenne muscular dystrophy. medRxiv preprint. 2024. 10.1101/2024.11.27.24318103. 10.1186/s12984-025-01718-5 PMC12486530 41034979

[17] Vandekerckhove I, Papageorgiou E, Hanssen B, De Beukelaer N, Van den Hauwe M, Goemans N, et al. Gait classification for growing children with Duchenne muscular dystrophy. Sci Rep. 2024;14:10828. 10.1038/s41598-024-61231-y. 38734731 10.1038/s41598-024-61231-y PMC11088636

[18] De Groote F, Falisse A. Perspective on musculoskeletal modelling and predictive simulations of human movement to assess the neuromechanics of gait. Proc Biol Sci. 2021;288:20202432. 10.1098/rspb.2020.2432. 10.1098/rspb.2020.2432 PMC7935082 33653141

[19] Ezati M, Ghannadi B, McPhee J. A review of simulation methods for human movement dynamics with emphasis on gait. Multibody Syst Dyn. 2019;47:265–92. 10.1007/s11044-019-09685-1.

[20] Falisse A, Serrancolí G, Dembia CL, Gillis J, Jonkers I, De Groote F. Rapid predictive simulations with complex musculoskeletal models suggest that diverse healthy and pathological human gaits can emerge from similar control strategies. J R Soc Interface. 2019;16:20190402. 10.1098/rsif.2019.0402. 31431186 10.1098/rsif.2019.0402 PMC6731507

[21] Falisse A, Afschrift M, De Groote F. Modeling toes contributes to realistic stance knee mechanics in three-dimensional predictive simulations of walking. PLoS ONE. 2022;17: e0256311. 10.1371/journal.pone.0256311. 35077455 10.1371/journal.pone.0256311 PMC8789163

[22] D’Hondt L, Falisse A, Gupta D, Van Den Bosch B, Buurke TJW, Febrer-Nafria M, et al. PredSim: A Framework for Rapid Predictive Simulations of Locomotion. 2024 10th IEEE RAS/EMBS International Conference for Biomedical Robotics and Biomechatronics (BioRob). 2024; pp. 1208-1213. 10.1109/BioRob60516.2024.10719735.

[23] D’Hondt L, De Groote F, Afschrift M. A dynamic foot model for predictive simulations of gait reveals causal relations between foot structure and whole body mechanics. bioRxiv Preprint. 2023. 10.1101/2023.03.22.533790. 10.1371/journal.pcbi.1012219 PMC11218950 38900787

[24] Falisse A, Pitto L, Kainz H, Hoang H, Wesseling M, Van Rossom S, et al. Physics-based simulations to predict the differential effects of motor control and musculoskeletal deficits on gait dysfunction in cerebral palsy: a retrospective case study. Front Hum Neurosci. 2020;14:40. 10.3389/fnhum.2020.00040. 32132911 10.3389/fnhum.2020.00040 PMC7040166

[25] D’Hondt L, De Groote F, Afschrift M. A dynamic foot model for predictive simulations of human gait reveals causal relations between foot structure and whole-body mechanics. PLoS Comput Biol. 2024;20: e1012219. 10.1371/journal.pcbi.1012219. 38900787 10.1371/journal.pcbi.1012219 PMC11218950

[26] Ong CF, Geijtenbeek T, Hicks JL, Delp SL. Predicting gait adaptations due to ankle plantarflexor muscle weakness and contracture using physics-based musculoskeletal simulations. PLoS Comput Biol. 2019;15: e1006993. 10.1371/journal.pcbi.1006993. 31589597 10.1371/journal.pcbi.1006993 PMC6797212

[27] Waterval N, Veerkamp K, Geijtenbeek T, Harlaar J, Nollet F, Brehm M, et al. Validation of forward simulations to predict the effects of bilateral plantarflexor weakness on gait. Gait Post. 2021;87:33–42. 10.1016/j.gaitpost.2021.04.020. 10.1016/j.gaitpost.2021.04.020 33882437

[28] Vandekerckhove I, Van den Hauwe M, De Beukelaer N, Stoop E, Goudriaan M, Delporte M, et al. Longitudinal alterations in gait features in growing children with Duchenne muscular dystrophy. Front Hum Neurosci. 2022;16: 861136. 10.3389/fnhum.2022.861136. 35721358 10.3389/fnhum.2022.861136 PMC9201072

[29] Vandekerckhove I, Van den Hauwe M, Dewit T, Molenberghs G, Goemans N, De Waele L, et al. Longitudinal trajectories of muscle impairments in growing boys with Duchenne muscular dystrophy. PLoS ONE. 2025;20: e0307007. 10.1371/journal.pone.0307007. 40100909 10.1371/journal.pone.0307007 PMC11918350

[30] Goudriaan M, Nieuwenhuys A, Schless S, Goemans N, Molenaers G, Desloovere K. A new strength assessment to evaluate the association between muscle weakness and gait pathology in children with cerebral palsy. PLoS ONE. 2018;13: e0191097. 10.1371/journal.pone.0191097. 29324873 10.1371/journal.pone.0191097 PMC5764363

[31] Verreydt I, Vandekerckhove I, Stoop E, Peeters N, van Tittelboom V, Van de Walle P, et al. Instrumented strength assessment in typically developing children and children with a neural or neuromuscular disorder: a reliability, validity and responsiveness study. Front Physiol. 2022;13: 855222. 10.3389/fphys.2022.855222. 36338500 10.3389/fphys.2022.855222 PMC9627606

[32] Vandekerckhove I, Hanssen B, Peeters N, Dewit T, De Beukelaer N, Van den Hauwe M, et al. Anthropometric-related percentile curves for muscle size and strength of lower limb muscles of typically developing children. Journal of Anatomy. 2025;00:1-15. 10.1111/joa.14241. 10.1111/joa.14241 PMC12265039 40098309

[33] Mudge AJ, Bau KV, Purcell LN, Wu JC, Axt MW, Selber P, et al. Normative reference values for lower limb joint range, bone torsion, and alignment in children aged 4–16 years. J Pediatr Orthop Part B. 2014;23:15–25. 10.1097/BPB.0b013e328364220a. 10.1097/BPB.0b013e328364220a PMC13007707 23852035

[34] Sankar WN, Laird CT, Baldwin KD. Hip range of motion in children: What is the norm? J Pediatr Orthop. 2012;32:399–405. 10.1097/BPO.0b013e3182519683. 22584842 10.1097/BPO.0b013e3182519683

[35] Ashworth B. Preliminary trial of carisoprodal in multiple sclerosis. Practitioner. 1964;192:540–2. 14143329

[36] Bohannon RW, Smith MB. Interrater reliability of a modified Ashworth scale of muscle spasticity. Phys Ther. 1987;67:206–7. 10.1093/ptj/67.2.206. 3809245 10.1093/ptj/67.2.206

[37] Hislop H, Avers D, Brown M. Muscle testing techniques of manual examination. 9th ed. Philadelphia: Elsevier; 1995.

[38] Anderson F, Pandy M. Dynamic optimization of human walking. J Biomech Eng. 2001;123:381–90. 10.1115/1.1392310. 11601721 10.1115/1.1392310

[39] Panjabi M, Oxland T, Yamamoto I, Crisco J. Mechanical behavior of the human lumbar and lumbosacral spine as shown by three-dimensional load-displacement curves. J Bone Jt Surg. 1994;76:413–24. 10.2106/00004623-199403000-00012. 10.2106/00004623-199403000-00012 8126047

[40] Raasch C, Zajac F, Ma B, Levine W. Muscle coordination of movement of maximum-speed pedaling. J Biomech. 1997;30:595–602. 10.1016/s0021-9290(96)00188-1. 9165393 10.1016/s0021-9290(96)00188-1

[41] De Groote F, Pipeleers G, Jonkers I, Demeulenaere B, Patten C, Swevers J, et al. A physiology based inverse dynamic analysis of human gait: Potential and perspectives. Comput Methods Biomech Biomed Engin. 2009;12:563–74. 10.1080/10255840902788587. 19319704 10.1080/10255840902788587

[42] Zajac FE. Muscle and tendon: properties, models, scaling, and application to biomechanics and motor control. Crit Rev Biomed Eng. 1989;17:359–411. 2676342

[43] De Groote F, Kinney AL, Rao AV, Fregly BJ. Evaluation of direct collocation optimal control problem formulations for solving the muscle redundancy problem. Ann Biomed Eng. 2016;44:2922–36. 10.1007/s10439-016-1591-9. 27001399 10.1007/s10439-016-1591-9 PMC5043004

[44] Falisse A, Serrancolí G, Dembia CL, Gillis J, De Groote F. Algorithmic differentiation improves the computational efficiency of OpenSim-based trajectory optimization of human movement. PLoS ONE. 2019;14: e0217730. 10.1371/journal.pone.0217730. 31622352 10.1371/journal.pone.0217730 PMC6797126

[45] Sherman MA, Seth A, Delp SL. Simbody: multibody dynamics for biomedical research. Proc IUTAM. 2011;2:241–61. 10.1016/j.piutam.2011.04.023. 10.1016/j.piutam.2011.04.023 PMC4390141 25866705

[46] Darras BT, Urion DK, Ghosh PS, et al. Dystrophinopathies. In: Adam MP, Feldman J, Mirzaa GM, et al., editors. GeneReviews®. Seattle (WA): University of Washington; 2000; pp. 1993–2025. https://www.ncbi.nlm.nih.gov/books/NBK1119/. 20301298

[47] Bhargava LJ, Pandy MG, Anderson FC. A phenomenological model for estimating metabolic energy consumption in muscle contraction. J Biomech. 2004;37:81–8. 10.1016/s0021-9290(03)00239-2. 14672571 10.1016/s0021-9290(03)00239-2

[48] Andersson JAE, Gillis J, Horn G, Rawlings JB, Diehl M. CasADi: a software framework for nonlinear optimization and optimal control. Math Progr Comput. 2019;11:1–36. 10.1007/s12532-018-0139-4.

[49] Wächter A, Biegler LT. On the implementation of an interior-point filter line-search algorithm for large-scale nonlinear programming. Math Program. 2006;106:25–57. 10.1007/s10107-004-0559-y.

[50] Kainz H, Goudriaan M, Falisse A, Huenaerts C, Desloovere K, De Groote F, et al. The influence of maximum isometric muscle force scaling on estimated muscle forces from musculoskeletal models of children with cerebral palsy. Gait Post. 2018;65:213–20. 10.1016/j.gaitpost.2018.07.172. 10.1016/j.gaitpost.2018.07.172 30558934

[51] Delp SL, Anderson FC, Arnold AS, Loan P, Habib A, John CT, et al. OpenSim: Open-source software to create and analyze dynamic simulations of movement. IEEE Trans Biomed Eng. 2007;54:1940–50. 10.1109/TBME.2007.901024. 18018689 10.1109/TBME.2007.901024

[52] Kainz H, Mindler GT, Kranzl A. Influence of femoral anteversion angle and neck-shaft angle on muscle forces and joint loading during walking. PLoS ONE. 2023;18: e0291458. 10.1371/journal.pone.0291458. 37824447 10.1371/journal.pone.0291458 PMC10569567

[53] Vandekerckhove I, Wesseling M, Kainz H, Desloovere K, Jonkers I. The effect of hip muscle weakness and femoral bony deformities on gait performance. Gait Post. 2021;83:280–6. 10.1016/j.gaitpost.2020.10.022. 10.1016/j.gaitpost.2020.10.022 33227606

[54] Wakeling JM, Febrer-Nafría M, De Groote F. A review of the efforts to develop muscle and musculoskeletal models for biomechanics in the last 50 years. J Biomech. 2023;155:111657. 10.1016/j.jbiomech.2023.111657. 37285780 10.1016/j.jbiomech.2023.111657

[55] Konno RN, Nigam N, Wakeling JM, Ross SA. The contributions of extracellular matrix and sarcomere properties to passive muscle stiffness in cerebral palsy. Front Physiol. 2022;12: 804188. 10.3389/fphys.2021.804188. 35153814 10.3389/fphys.2021.804188 PMC8827041

[56] Virgilio KM, Martin KS, Peirce SM, Blemker SS. Multiscale models of skeletal muscle reveal the complex effects of muscular dystrophy on tissue mechanics and damage susceptibility. Interface Focus. 2015;5:20140080. 10.1098/rsfs.2014.0080. 25844152 10.1098/rsfs.2014.0080 PMC4342948

[57] Zebracki K, Drotar D. Pain and activity limitations in children with Duchenne or Becker muscular dystrophy. Dev Med Child Neurol. 2008;50:546–52. 10.1111/j.1469-8749.2008.03005.x. 18611207 10.1111/j.1469-8749.2008.03005.x

[58] Kainz H, Modenese L, Lloyd DG, Maine S, Walsh HPJ, Carty CP. Joint kinematic calculation based on clinical direct kinematic versus inverse kinematic gait models. J Biomech. 2016;49:1658–69. 10.1016/j.jbiomech.2016.03.052. 27139005 10.1016/j.jbiomech.2016.03.052

