# Supplementary material for: Muscle weakness but also contractures contribute to the progressive gait pathology in children with Duchenne muscular dystrophy: a simulation study
Source: J Neuroeng Rehabil. 2025 May 4;22:103. doi: 10.1186/s12984-025-01631-x (PMC12051353; doi:10.1186/s12984-025-01631-x)
Supplement: Supplementary file 1 — Additional file 1. [file 12984_2025_1631_MOESM1_ESM.pdf]

Additional file 1: Details on scaling of generic musculoskeletal model to child's dimensions

The maximal isometric muscle forces (F_m^{max})[1] and ligament forces (F_{lig}^{pcsa})[2] were scaled to the expected TD body mass for the DMD groups and the mean body mass of the group for the TD group (Eq. 1 and Eq. 2):

$$F_m^{max} = F_{m,gen}^{max} * \left(\frac{M_{(e)TD}}{M_{gen}}\right)^{\frac{2}{3}} \quad (1)$$

$$F_{lig}^{pcsa} = F_{lig,gen}^{pcsa} * \left(\frac{M_{(e)TD}}{M_{gen}}\right)^{\frac{2}{3}} \quad (2)$$

where *gen* refers to the generic musculoskeletal model of D'Hondt et al.[2], M_{gen} refers to body mass of the generic model, $M_{(e)TD}$ refers to the expected mean body mass of TD children of the same length as the mean length of the DMD group (M_{eTD}) or the mean body mass of the TD group (M_{TD}).

The stiffness coefficients (K_{pass}) and damping (d_{pass}) of the passive joint torques were scaled to the expected TD body mass for the DMD groups and the mean body mass of the group for the TD group, and the mean length of the group (Eq. 3 and Eq. 4):

$$K_{pass} = K_{pass,gen} * \left(\frac{M_{(e)TD}*L}{M_{gen}*L_{gen}}\right) \quad (3)$$

$$d_{pass} = d_{pass,gen} * \left(\frac{M_{(e)TD}*L}{M_{gen}*L_{gen}}\right) \quad (4)$$

where *gen* refers to the generic musculoskeletal model of D'Hondt et al.[2], M_{gen} refers to body mass of the generic model, L_{gen} refers to length of generic model, L refers to mean length of the specific group, $M_{(e)TD}$ refers to the expected mean body mass of TD children of the same length as the mean length of the DMD group (M_{eTD}) or the mean body mass of the TD group (M_{TD}).

The location (c_{CS}), size (r_{CS}), and damping (d_{CS}) of the contact spheres were scaled to the mean length of the group (Eq. 5, Eq. 6 and Eq. 7):

$$c_{CS} = c_{CS,gen} * \left(\frac{L}{L_{gen}}\right) \tag{5}$$

$$r_{CS} = r_{CS,gen} * \left(\frac{L}{L_{gen}}\right) \tag{6}$$

$$d_{CS} = d_{CS,gen} * \left(\frac{L}{L_{gen}}\right) \tag{7}$$

where *gen* refers to the generic musculoskeletal model of D'Hondt et al.[2], L_{gen} refers to length of generic model, L refers to mean length of the specific group.

The stiffness (K_{CS}) of the contact spheres were scaled to the expected TD body mass for the DMD groups and the mean body mass of the group for the TD group, and the mean length of the group (Eq. 8):

$$K_{CS} = K_{CS,gen} * \left(\frac{M_{(e)TD}}{M_{gen}} * \left(\frac{L_{gen}}{L}\right)^2\right) \tag{8}$$

where *gen* refers to the generic musculoskeletal model of D'Hondt et al.[2], M_{gen} refers to body mass of the generic model, L_{gen} refers to length of generic model, L refers to mean length of the specific group, $M_{(e)TD}$ refers to the expected mean body mass of TD children of the same length as the mean length of the DMD group (M_{eTD}) or the mean body mass of the TD group (M_{TD}).

References:

1. van der Krogt MM, Bar-On L, Kindt T, Desloovere K, Harlaar J. Neuro-musculoskeletal simulation of instrumented contracture and spasticity assessment in children with cerebral palsy. J Neuroeng Rehabil [Internet]. 2016;13:64. Available from: http://jneuroengrehab.biomedcentral.com/articles/10.1186/s12984-016-0170-5

2. D'Hondt L, De Groote F, Afschrift M. A dynamic foot model for predictive simulations of gait reveals causal relations between foot structure and whole body mechanics. bioRxiv Prepr [Internet]. 2023; Available from: doi: https://doi.org/10.1101/2023.03.22.533790
