# Supplementary material for: Muscle weakness but also contractures contribute to the progressive gait pathology in children with Duchenne muscular dystrophy: a simulation study
Source: J Neuroeng Rehabil. 2025 May 4;22:103. doi: 10.1186/s12984-025-01631-x (PMC12051353; doi:10.1186/s12984-025-01631-x)
Supplement: Supplementary file 2 — Additional file 2. [file 12984_2025_1631_MOESM2_ESM.pdf]

Additional file 2: Details on scaling predictive simulations to child's dimensions

Distance constraints ($dB_{seg}^{low,up}$) were scaled to the body length:

$$dB_{seg}^{low,up} = dB_{seg,gen}^{low,up} * \left(\frac{L}{L_{gen}}\right) \quad (1)$$

where *gen* refers to the generic musculoskeletal model of D'Hondt et al.[1], L_{gen} refers to length of generic model, L refers to mean length of the specific group.

The weight factor w_1 and w_4 were scaled to body length and body mass:

$$w_1 = \frac{w_{1,gen}}{\frac{M_{DMD,TD}}{M_{gen}} * \left(\frac{L}{L_{gen}}\right)^2} \quad (2)$$

$$w_4 = \frac{w_{4,gen}}{\left(\frac{M_{(e)TD} * L}{M_{gen} * L_{gen}}\right)^2} \quad (3)$$

where *gen* refers to the generic musculoskeletal model of D'Hondt et al.[1], M_{gen} refers to body mass of the generic model, L_{gen} refers to length of generic model, L refers to mean length of the specific group, $M_{DMD,TD}$ refers to the mean body mass of the DMD group or TD group, $M_{(e)TD}$ refers to the expected mean body mass of TD children of the same length as the mean length of the DMD group (M_{eTD}) or the mean body mass of the TD group (M_{TD}).

References:

1. D'Hondt L, De Groote F, Afschrift M. A dynamic foot model for predictive simulations of gait reveals causal relations between foot structure and whole body mechanics. bioRxiv Prepr [Internet]. 2023; Available from: doi: https://doi.org/10.1101/2023.03.22.533790
