# Supplementary material for: Muscle weakness but also contractures contribute to the progressive gait pathology in children with Duchenne muscular dystrophy: a simulation study
Source: J Neuroeng Rehabil. 2025 May 4;22:103. doi: 10.1186/s12984-025-01631-x (PMC12051353; doi:10.1186/s12984-025-01631-x)
Supplement: Supplementary file 3 — Additional file 3. [file 12984_2025_1631_MOESM3_ESM.pdf]

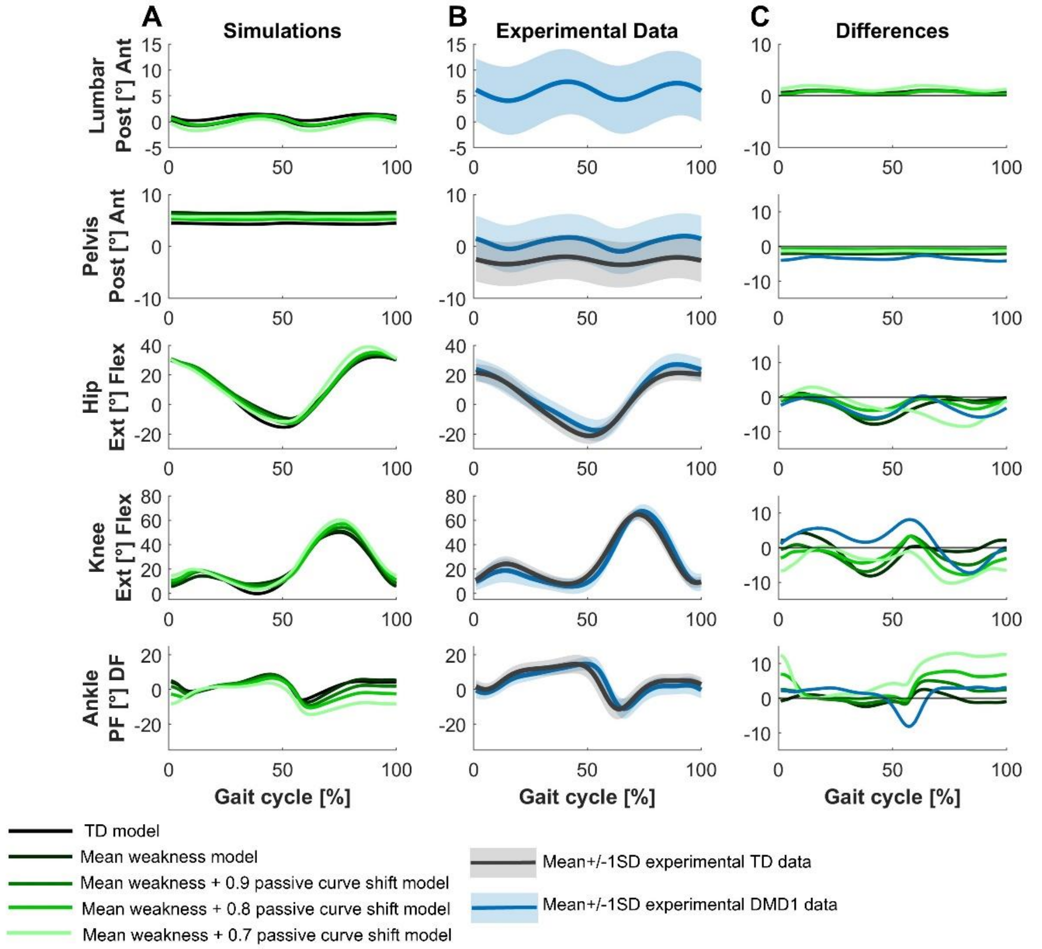

Figure S1: *Sensitivity analysis of progressively increasing contractures by shifting the passive force-length curve to 0.9, 0.8 and 0.7 of the normalized fiber length while holding muscle weakness constant at its mean value on sagittal plane kinematics for DMD1.* ***A.*** *Simulated kinematics* ***B.*** *Experimental gait kinematics.* ***C.*** *Differences in kinematics between the TD model and the mean weakness model (very dark green), the TD model and the mean weakness with the passive force-length curve at 0.9 of the normalized fiber length model (dark green), the TD model and the mean weakness with the passive force-length curve at 0.8 of the normalized fiber length model (light green), the TD model and the mean weakness with the passive force-length curve at 0.7 of the normalized fiber length model (very light green), and the experimental TD and DMD1 data (blue). Abbreviations: Ant, anterior; DF, dorsiflexion; Ext, extension; Flex, flexion; PF, plantar flexion; Post, posterior; TD, typically developing;*

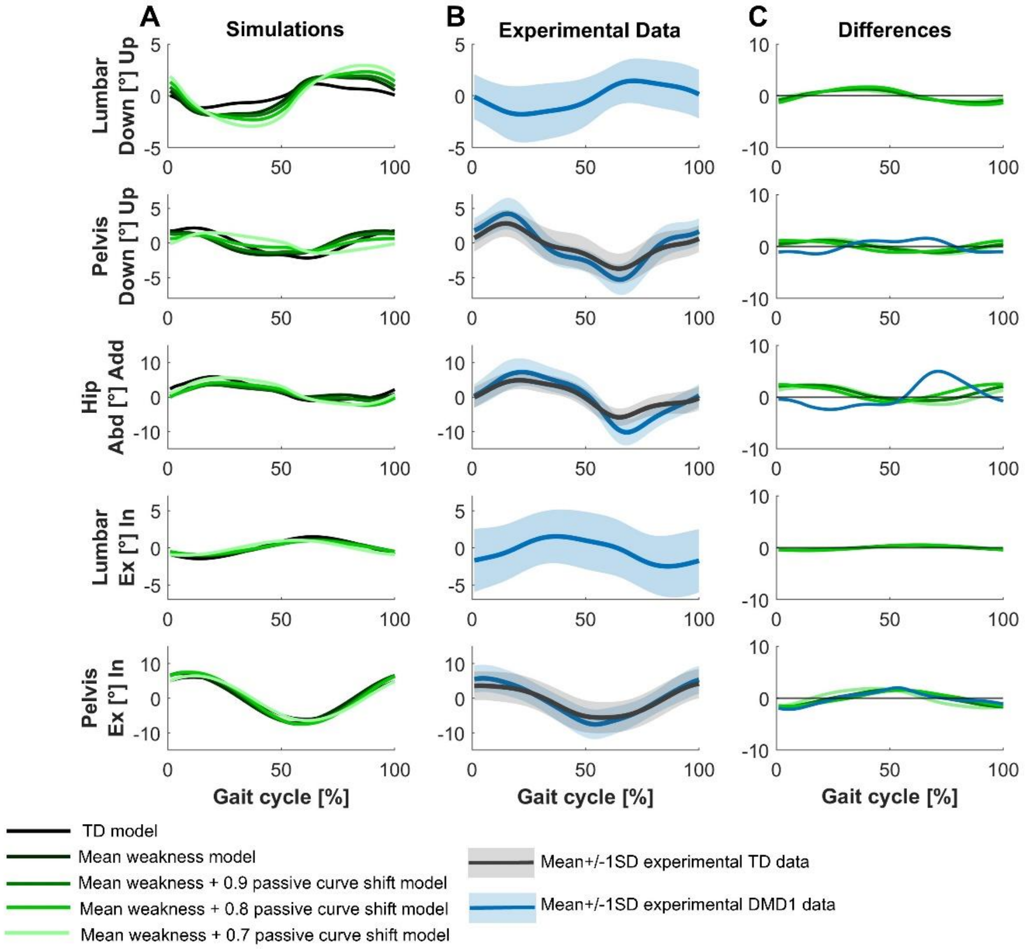

Figure S2: *Sensitivity analysis of progressively increasing contractures by shifting the passive force-length curve to 0.9, 0.8 and 0.7 of the normalized fiber length while holding muscle weakness constant at its mean value on frontal and transverse plane kinematics for DMD1.* ***A.*** *Simulated kinematics* ***B.*** *Experimental gait kinematics.* ***C.*** *Differences in kinematics between the TD model and the mean weakness model (very dark green), the TD model and the mean weakness with the passive force-length curve at 0.9 of the normalized fiber length model (dark green), the TD model and the mean weakness with the passive force-length curve at 0.8 of the normalized fiber length model (light green), the TD model and the mean weakness with the passive force-length curve at 0.7 of the normalized fiber length model (very light green), and the experimental TD and DMD1 data (blue). Abbreviations: Abd, abduction; Add, adduction; Ex, external; In, internal; TD, typically developing;*

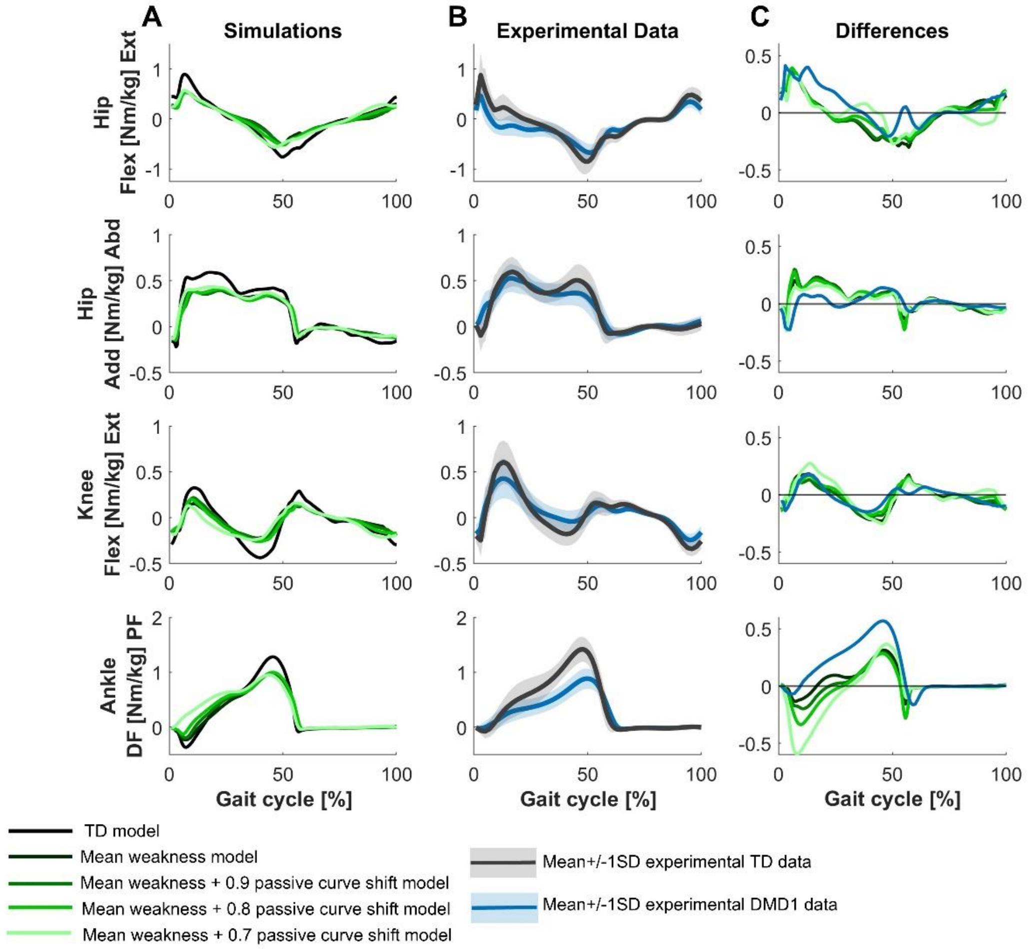

Figure S3: *Sensitivity analysis of progressively increasing contractures by shifting the passive force-length curve to 0.9, 0.8 and 0.7 of the normalized fiber length while holding muscle weakness constant at its mean value on kinetics for DMD1.* ***A.*** *Simulated kinetics* ***B.*** *Experimental gait kinetics.* ***C.*** *Differences in kinetics between the TD model and the mean weakness model (very dark green), the TD model and the mean weakness with the passive force-length curve at 0.9 of the normalized fiber length model (dark green), the TD model and the mean weakness with the passive force-length curve at 0.8 of the normalized fiber length model (light green), the TD model and the mean weakness with the passive force-length curve at 0.7 of the normalized fiber length model (very light green), and the experimental TD and DMD1 data (blue). Abbreviations: Abd, abduction; Add, adduction; DF, dorsiflexion; Ext, extension; Flex, flexion; PF, plantar flexion; TD, typically developing;*

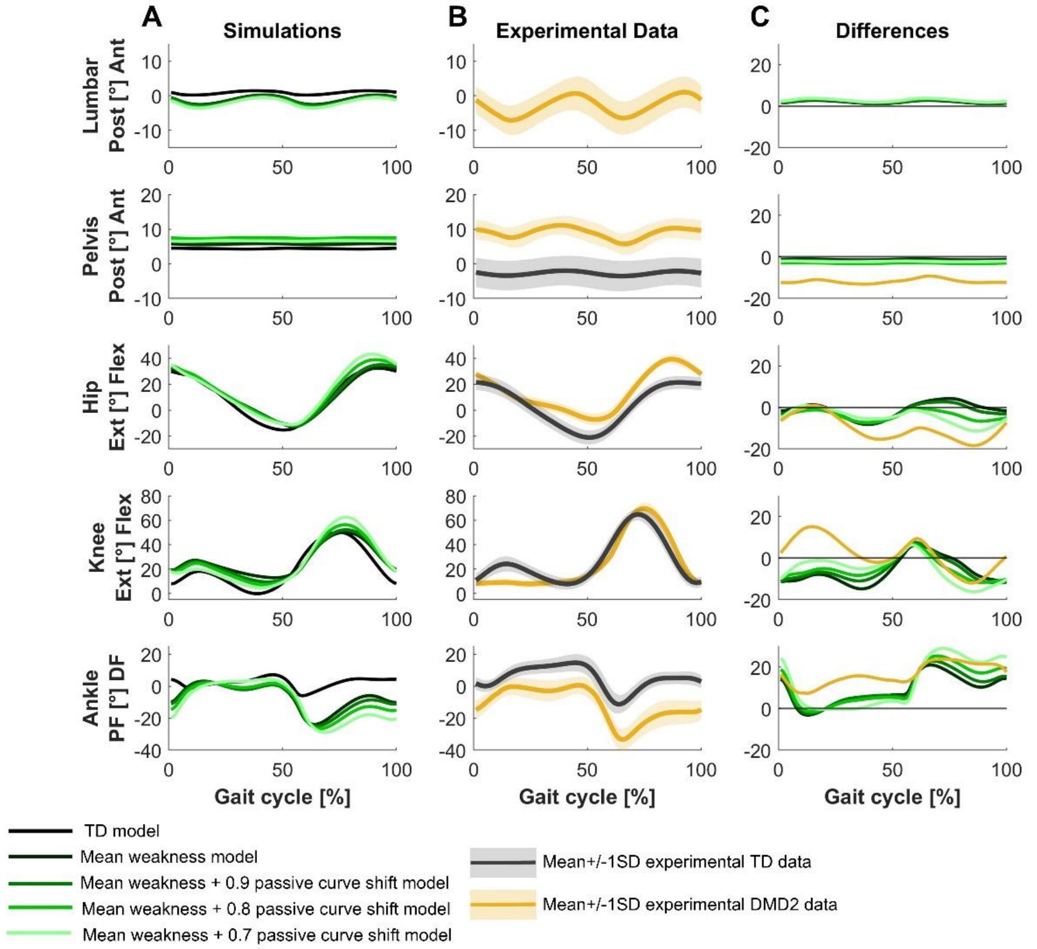

Figure S4: *Sensitivity analysis of progressively increasing contractures by shifting the passive force-length curve to 0.9, 0.8 and 0.7 of the normalized fiber length while holding muscle weakness constant at its mean value on sagittal plane kinematics for DMD2.* ***A.*** *Simulated kinematics* ***B.*** *Experimental gait kinematics.* ***C.*** *Differences in kinematics between the TD model and the mean weakness model (very dark green), the TD model and the mean weakness with the passive force-length curve at 0.9 of the normalized fiber length model (dark green), the TD model and the mean weakness with the passive force-length curve at 0.8 of the normalized fiber length model (light green), the TD model and the mean weakness with the passive force-length curve at 0.7 of the normalized fiber length model (very light green), and the experimental TD and DMD2 data (yellow). Abbreviations: Ant, anterior; DF, dorsiflexion; Ext, extension; Flex, flexion; PF, plantar flexion; Post, posterior; TD, typically developing;*

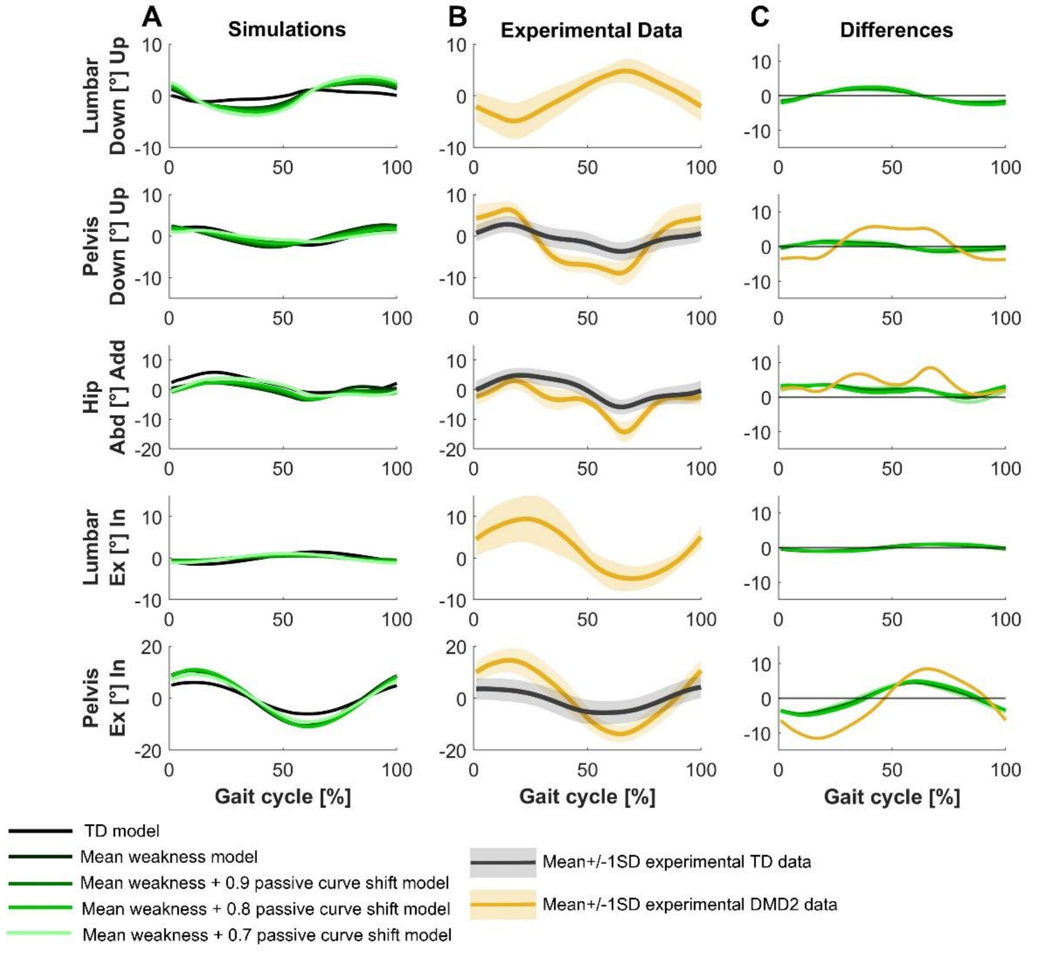

Figure S5: *Sensitivity analysis of progressively increasing contractures by shifting the passive force-length curve to 0.9, 0.8 and 0.7 of the normalized fiber length while holding muscle weakness constant at its mean value on frontal and transverse plane kinematics for DMD2.* ***A.*** *Simulated kinematics* ***B.*** *Experimental gait kinematics.* ***C.*** *Differences in kinematics between the TD model and the mean weakness model (very dark green), the TD model and the mean weakness with the passive force-length curve at 0.9 of the normalized fiber length model (dark green), the TD model and the mean weakness with the passive force-length curve at 0.8 of the normalized fiber length model (light green), the TD model and the mean weakness with the passive force-length curve at 0.7 of the normalized fiber length model (very light green), and the experimental TD and DMD2 data (yellow). Abbreviations: Abd, abduction; Add, adduction; Ex, external; In, internal; TD, typically developing;*

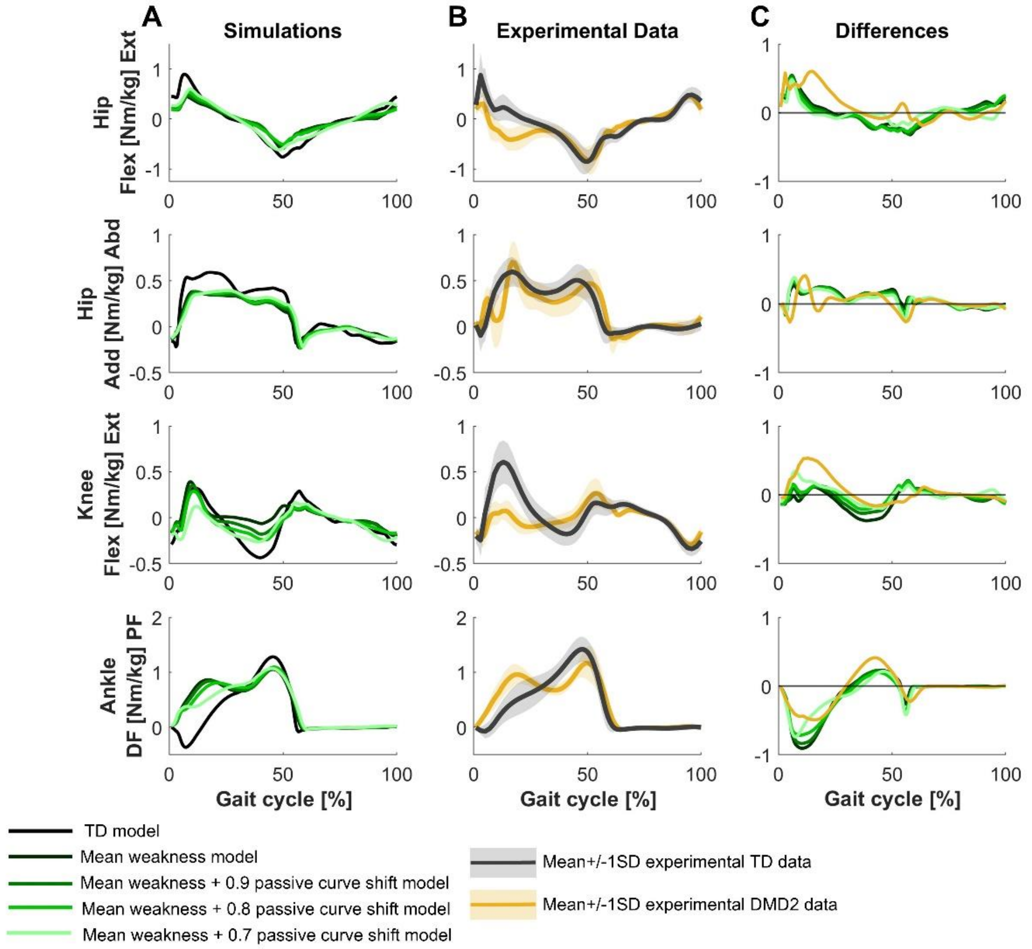

Figure S6: *Sensitivity analysis of progressively increasing contractures by shifting the passive force-length curve to 0.9, 0.8 and 0.7 of the normalized fiber length while holding muscle weakness constant at its mean value on kinetics for DMD2.* ***A.*** *Simulated kinetics* ***B.*** *Experimental gait kinetics.* ***C.*** *Differences in kinetics between the TD model and the mean weakness model (very dark green), the TD model and the mean weakness with the passive force-length curve at 0.9 of the normalized fiber length model (dark green), the TD model and the mean weakness with the passive force-length curve at 0.8 of the normalized fiber length model (light green), the TD model and the mean weakness with the passive force-length curve at 0.7 of the normalized fiber length model (very light green), and the experimental TD and DMD2 data (yellow). Abbreviations: Abd, abduction; Add, adduction; DF, dorsiflexion; Ext, extension; Flex, flexion; PF, plantar flexion; TD, typically developing;*

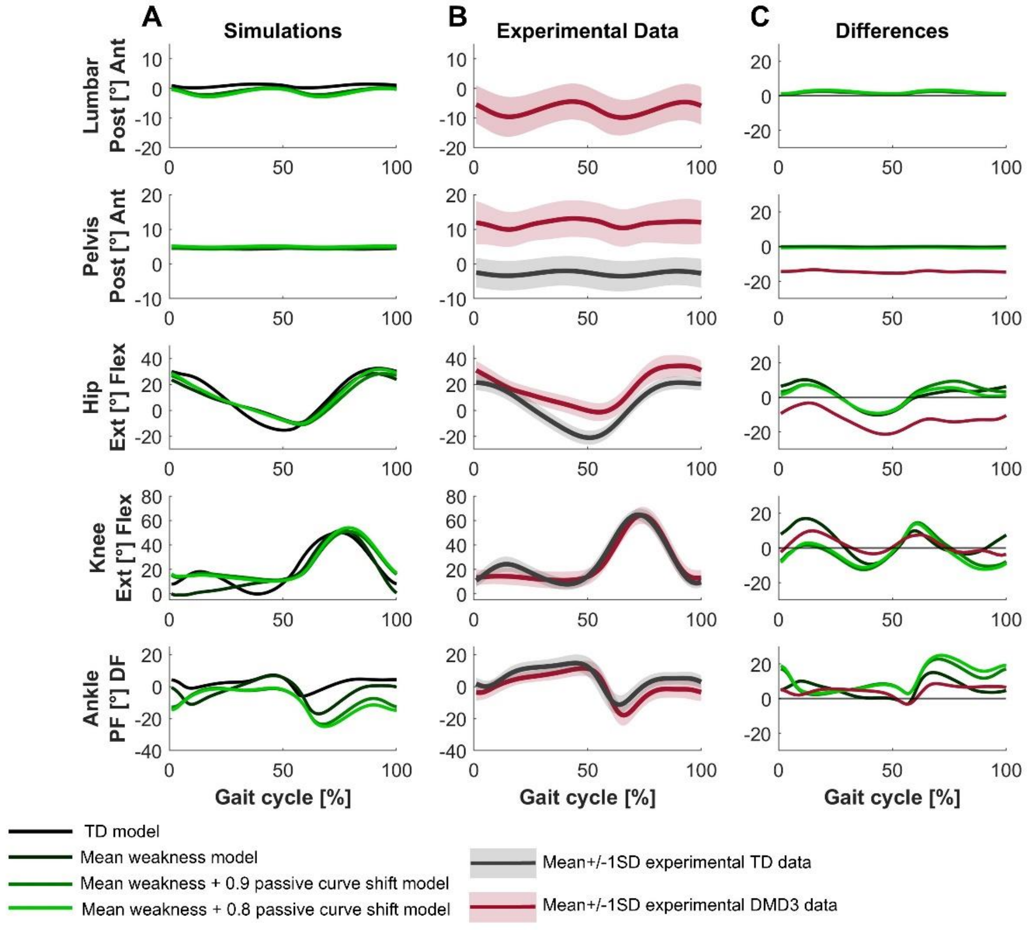

Figure S7: *Sensitivity analysis of progressively increasing contractures by shifting the passive force-length curve to 0.9, 0.8 and 0.7 of the normalized fiber length while holding muscle weakness constant at its mean value on sagittal plane kinematics for DMD3.* ***A.*** *Simulated kinematics* ***B.*** *Experimental gait kinematics.* ***C.*** *Differences in kinematics between the TD model and the mean weakness model (very dark green), the TD model and the mean weakness with the passive force-length curve at 0.9 of the normalized fiber length model (dark green), the TD model and the mean weakness with the passive force-length curve at 0.8 of the normalized fiber length model (light green), and the experimental TD and DMD3 data (red). The simulation based on the mean weakness with the passive force-length curve at 0.7 of the normalized fiber length model could not find a feasible solution for DMD3. Abbreviations: Ant, anterior; DF, dorsiflexion; Ext, extension; Flex, flexion; PF, plantar flexion; Post, posterior; TD, typically developing;*

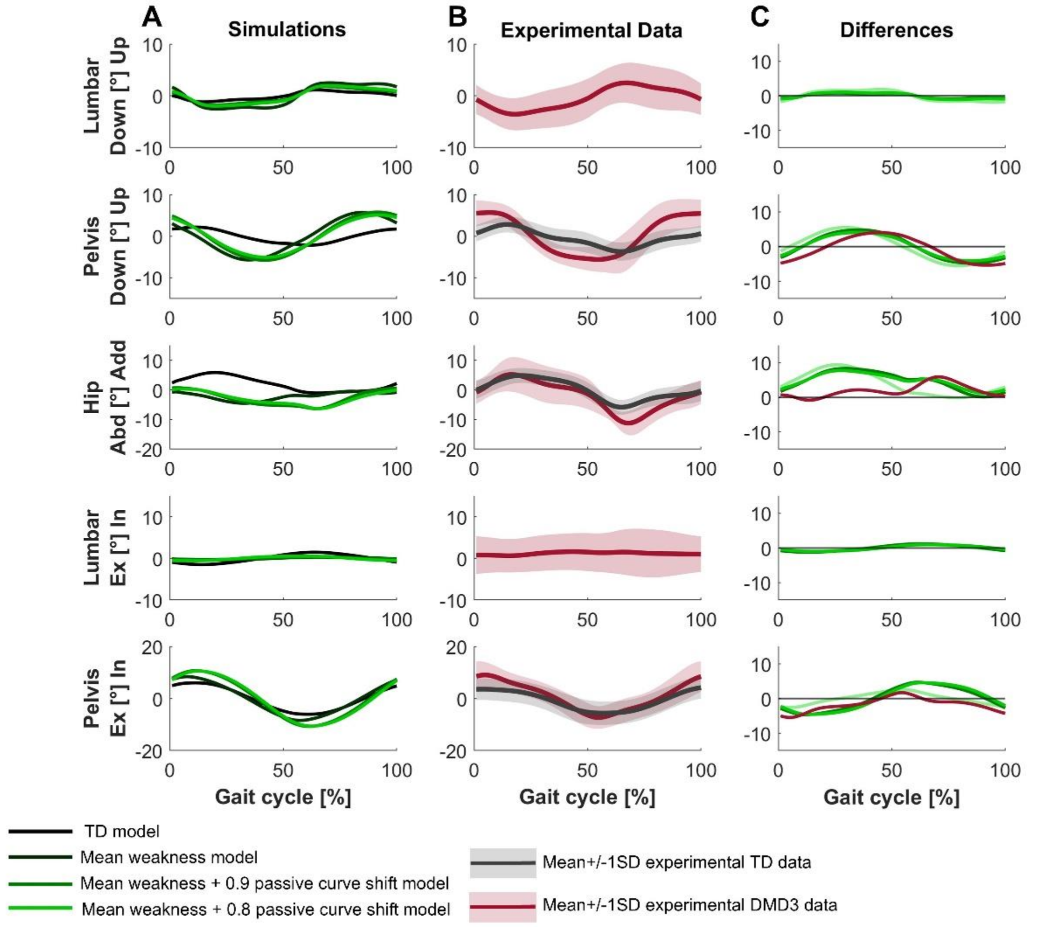

Figure S8: *Sensitivity analysis of progressively increasing contractures by shifting the passive force-length curve to 0.9, 0.8 and 0.7 of the normalized fiber length while holding muscle weakness constant at its mean value on frontal and transverse plane kinematics for DMD3.* ***A.*** *Simulated kinematics* ***B.*** *Experimental gait kinematics.* ***C.*** *Differences in kinematics between the TD model and the mean weakness model (very dark green), the TD model and the mean weakness with the passive force-length curve at 0.9 of the normalized fiber length model (dark green), the TD model and the mean weakness with the passive force-length curve at 0.8 of the normalized fiber length model (light green), and the experimental TD and DMD3 data (red). The simulation based on the mean weakness with the passive force-length curve at 0.7 of the normalized fiber length model could not find a feasible solution for DMD3. Abbreviations: Abd, abduction; Add, adduction; Ex, external; In, internal; TD, typically developing;*

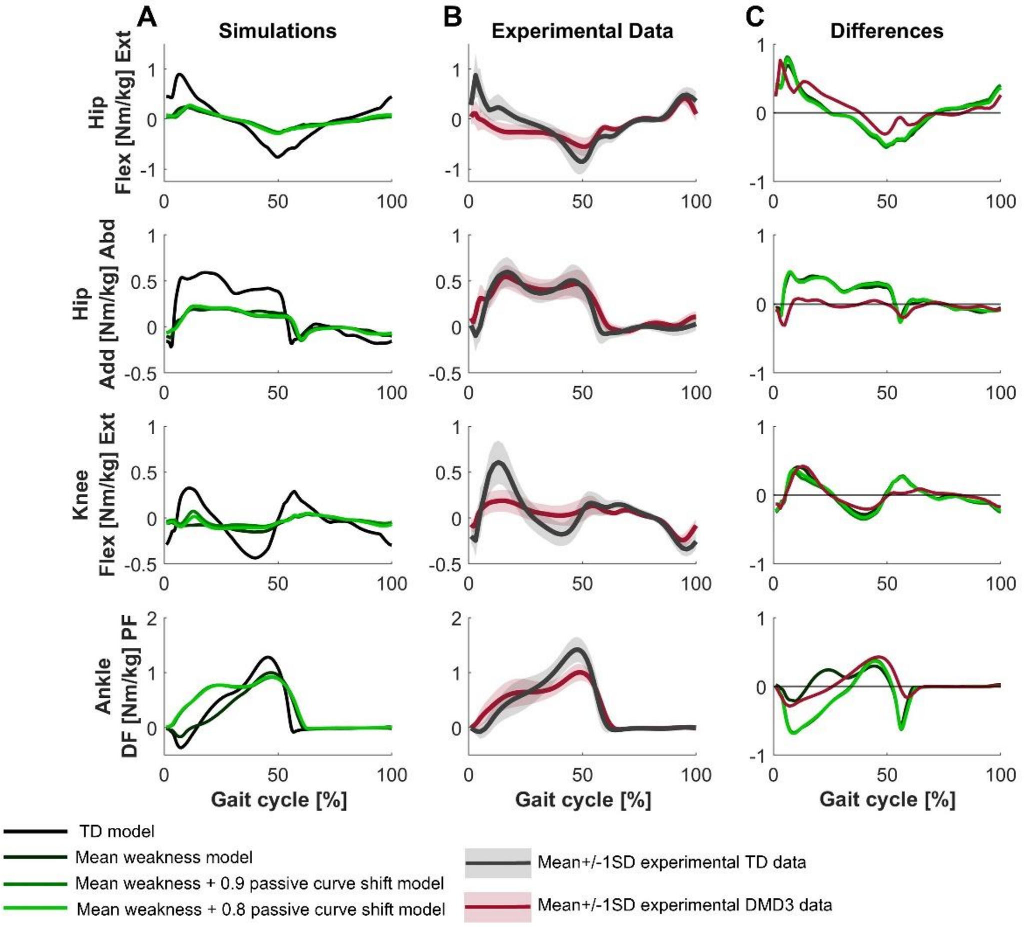

Figure S9: *Sensitivity analysis of progressively increasing contractures by shifting the passive force-length curve to 0.9, 0.8 and 0.7 of the normalized fiber length while holding muscle weakness constant at its mean value on kinetics for DMD3.* ***A.*** *Simulated kinetics* ***B.*** *Experimental gait kinetics.* ***C.*** *Differences in kinetics between the TD model and the mean weakness model (very dark green), the TD model and the mean weakness with the passive force-length curve at 0.9 of the normalized fiber length model (dark green), the TD model and the mean weakness with the passive force-length curve at 0.8 of the normalized fiber length model (light green), and the experimental TD and DMD3 data (red). The simulation based on the mean weakness with the passive force-length curve at 0.7 of the normalized fiber length model could not find a feasible solution for DMD3. Abbreviations: Abd, abduction; Add, adduction; DF, dorsiflexion; Ext, extension; Flex, flexion; PF, plantar flexion; TD, typically developing;*
