# Supplementary material for: Muscle weakness but also contractures contribute to the progressive gait pathology in children with Duchenne muscular dystrophy: a simulation study
Source: J Neuroeng Rehabil. 2025 May 4;22:103. doi: 10.1186/s12984-025-01631-x (PMC12051353; doi:10.1186/s12984-025-01631-x)
Supplement: Supplementary file 4 — Additional file 4. [file 12984_2025_1631_MOESM4_ESM.pdf]

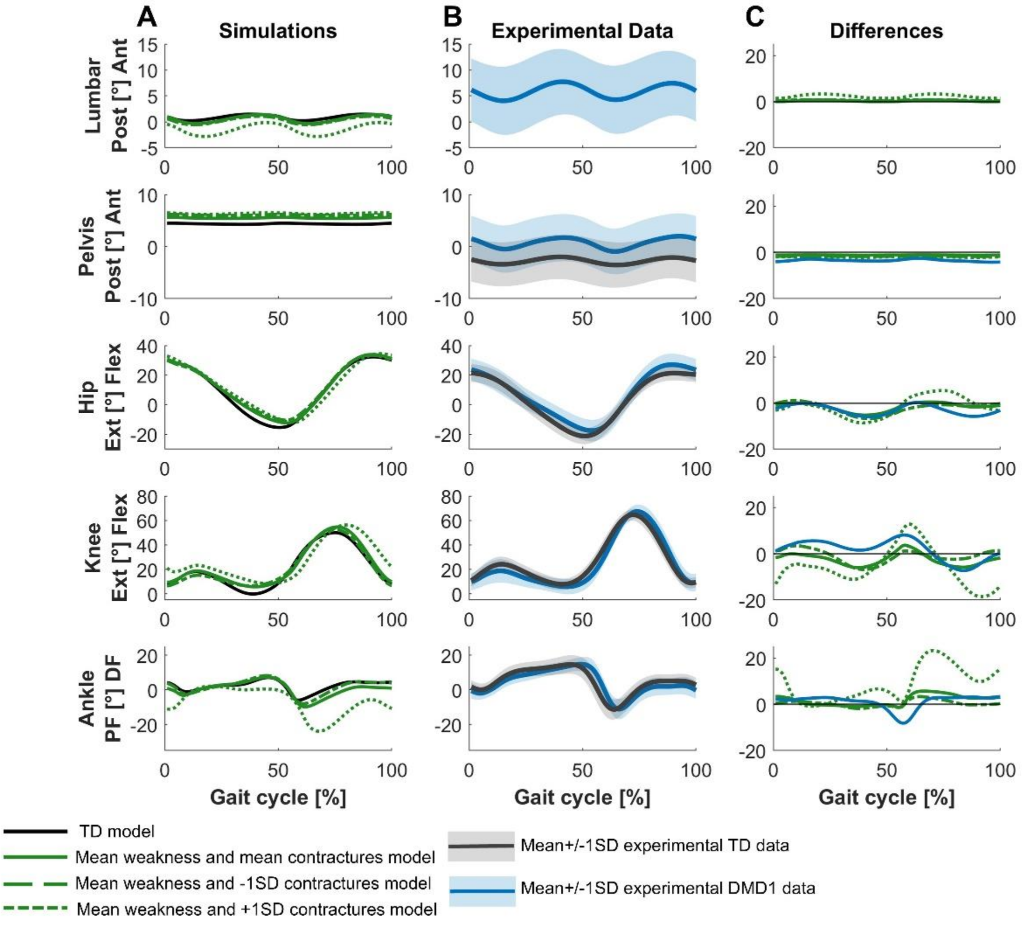

Figure S10: *Sensitivity analysis varying contractures around their mean value by ±1 SD while holding muscle weakness constant at its mean value on sagittal plane kinematics for DMD1.* ***A.*** *Simulated kinematics* ***B.*** *Experimental gait kinematics.* ***C.*** *Differences in kinematics between the TD model and the mean weakness + mean contractures model (solid line), the TD model and the mean weakness with -1SD contractures model (long dotted line), the TD model and the mean weakness with +1SD contractures model (short dotted line), and the experimental TD and DMD1 data (blue). Abbreviations: Ant, anterior; DF, dorsiflexion; Ext, extension; Flex, flexion; PF, plantar flexion; Post, posterior; TD, typically developing;*

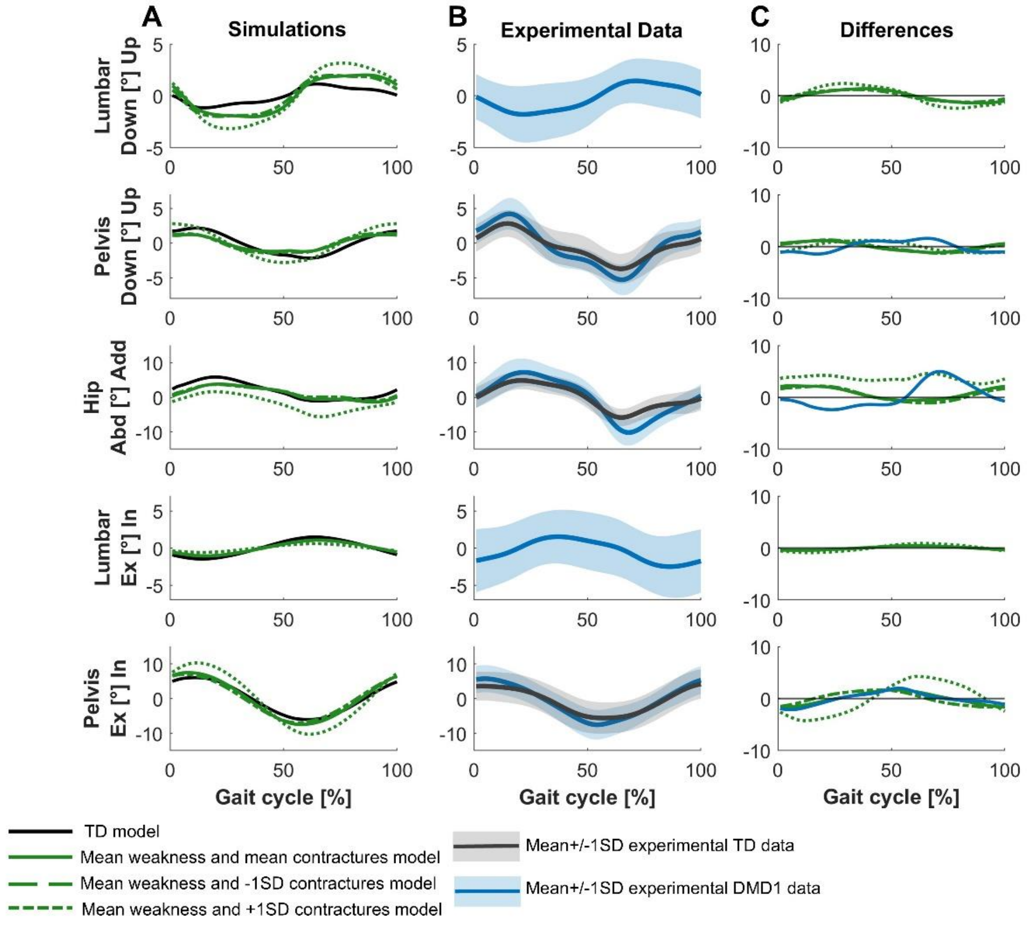

Figure S11: *Sensitivity analysis varying contractures around their mean value by ±1 SD while holding muscle weakness constant at its mean value on frontal and transverse plane kinematics for DMD1.* ***A.*** *Simulated kinematics* ***B.*** *Experimental gait kinematics.* ***C.*** *Differences in kinematics between the TD model and the mean weakness + mean contractures model (solid line), the TD model and the mean weakness with -1SD contractures model (long dotted line), the TD model and the mean weakness with +1SD contractures model (short dotted line), and the experimental TD and DMD1 data (blue). Abbreviations: Abd, abduction; Add, adduction; Ex, external; In, internal; TD, typically developing;*

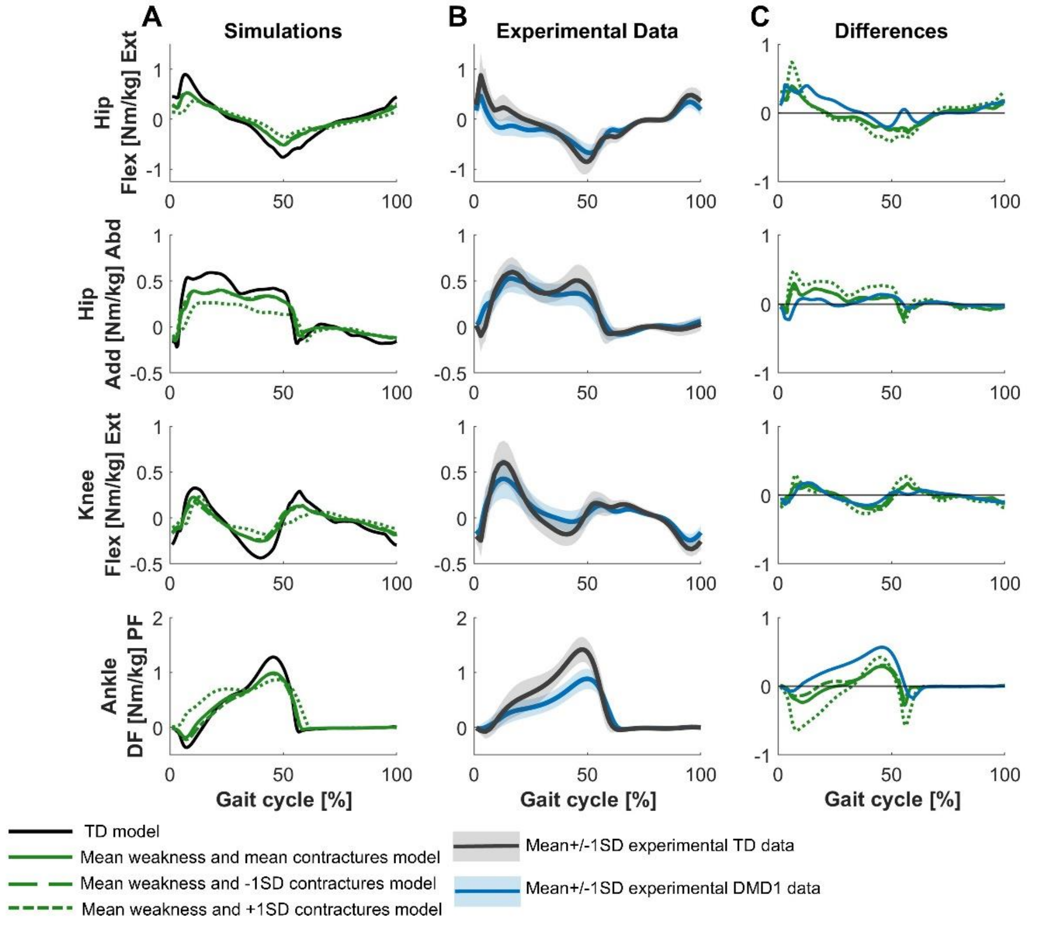

Figure S12: *Sensitivity analysis varying contractures around their mean value by ±1 SD while holding muscle weakness constant at its mean value on kinetics for DMD1.* ***A.*** *Simulated kinetics* ***B.*** *Experimental gait kinetics.* ***C.*** *Differences in kinetics between the TD model and the mean weakness + mean contractures model (solid line), the TD model and the mean weakness with -1SD contractures model (long dotted line), the TD model and the mean weakness with +1SD contractures model (short dotted line), and the experimental TD and DMD1 data (blue). Abbreviations: Abd, abduction; Add, adduction; DF, dorsiflexion; Ext, extension; Flex, flexion; PF, plantar flexion; TD, typically developing;*

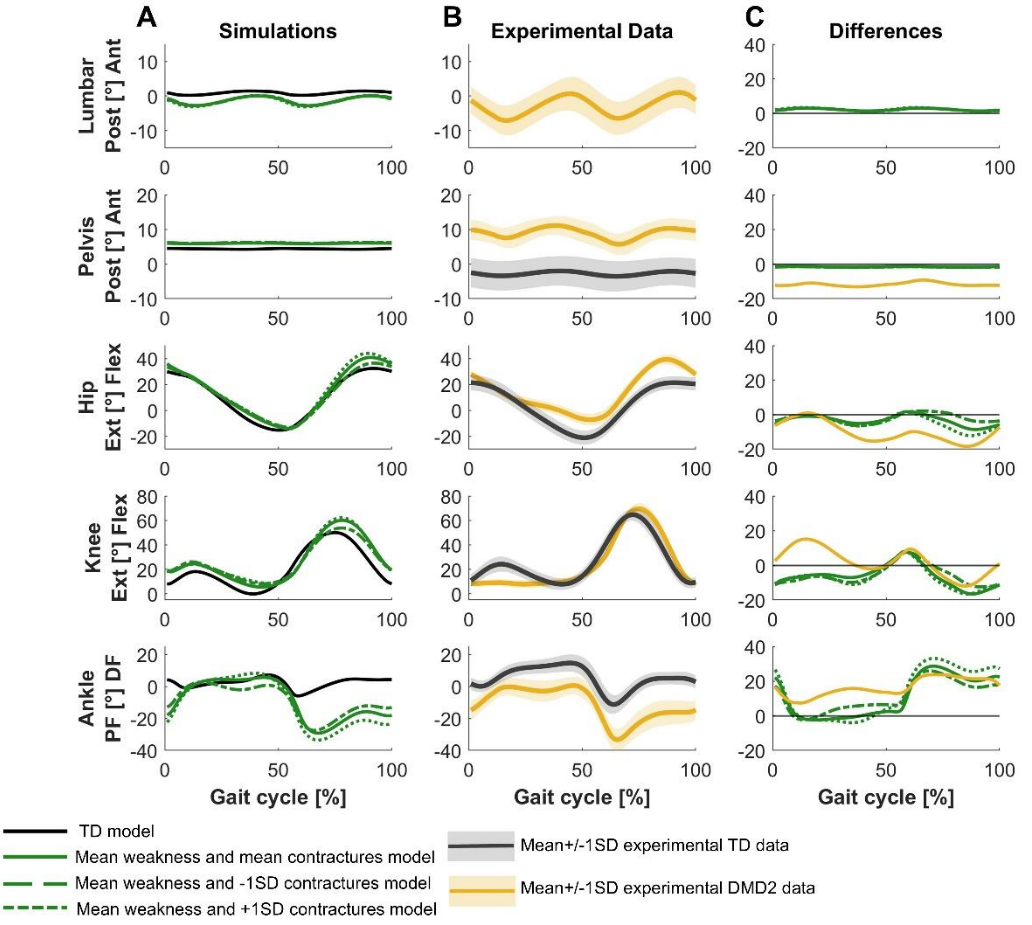

Figure S13: *Sensitivity analysis varying contractures around their mean value by ±1 SD while holding muscle weakness constant at its mean value on sagittal plane kinematics for DMD2.* ***A.*** *Simulated kinematics* ***B.*** *Experimental gait kinematics.* ***C.*** *Differences in kinematics between the TD model and the mean weakness + mean contractures model (solid line), the TD model and the mean weakness with -1SD contractures model (long dotted line), the TD model and the mean weakness with +1SD contractures model (short dotted line), and the experimental TD and DMD2 data (yellow). Abbreviations: Ant, anterior; DF, dorsiflexion; Ext, extension; Flex, flexion; PF, plantar flexion; Post, posterior; TD, typically developing;*

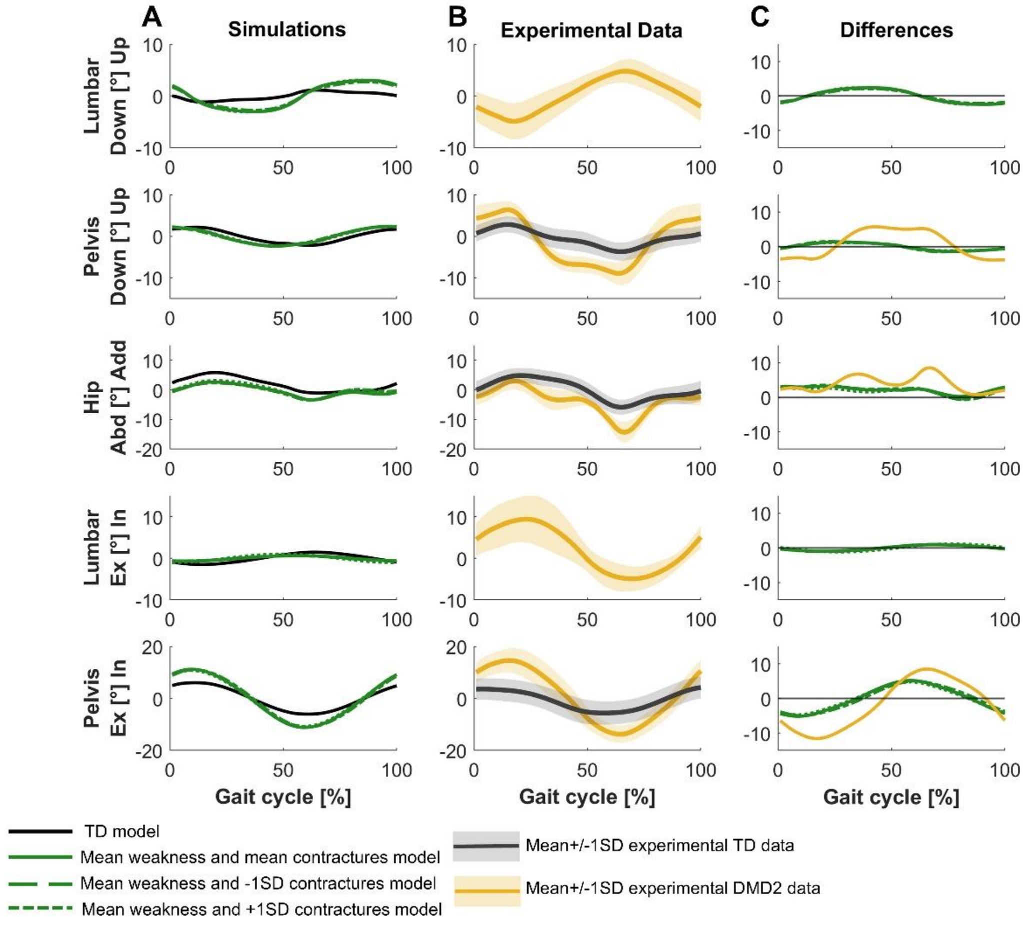

Figure S14: *Sensitivity analysis varying contractures around their mean value by ±1 SD while holding muscle weakness constant at its mean value on frontal and transverse plane kinematics for DMD2.* ***A.*** *Simulated kinematics* ***B.*** *Experimental gait kinematics.* ***C.*** *Differences in kinematics between the TD model and the mean weakness + mean contractures model (solid line), the TD model and the mean weakness with -1SD contractures model (long dotted line), the TD model and the mean weakness with +1SD contractures model (short dotted line), and the experimental TD and DMD2 data (yellow). Abbreviations: Abd, abduction; Add, adduction; Ex, external; In, internal; TD, typically developing;*

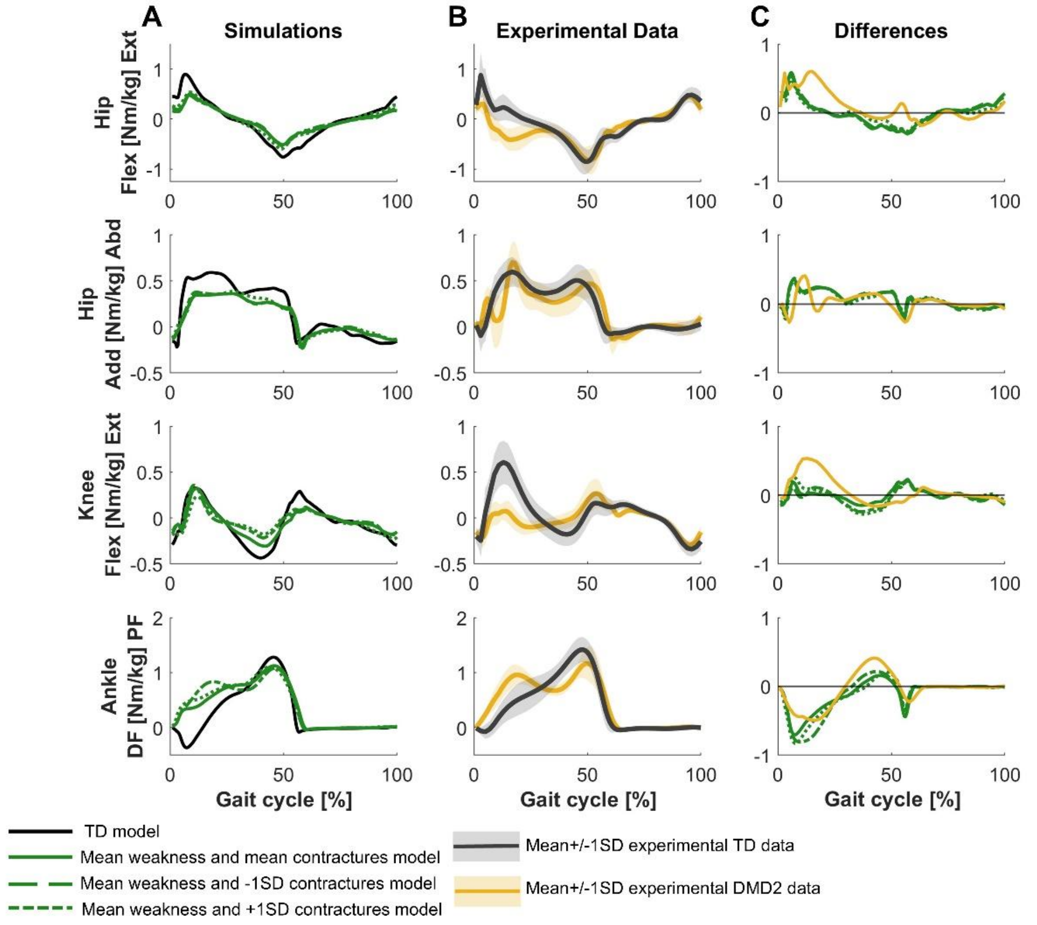

Figure S15: *Sensitivity analysis varying contractures around their mean value by ±1 SD while holding muscle weakness constant at its mean value on kinetics for DMD2.* ***A.*** *Simulated kinetics* ***B.*** *Experimental gait kinetics.* ***C.*** *Differences in kinetics between the TD model and the mean weakness + mean contractures model (solid line), the TD model and the mean weakness with -1SD contractures model (long dotted line), the TD model and the mean weakness with +1SD contractures model (short dotted line), and the experimental TD and DMD2 data (yellow). Abbreviations: Abd, abduction; Add, adduction; DF, dorsiflexion; Ext, extension; Flex, flexion; PF, plantar flexion; TD, typically developing;*

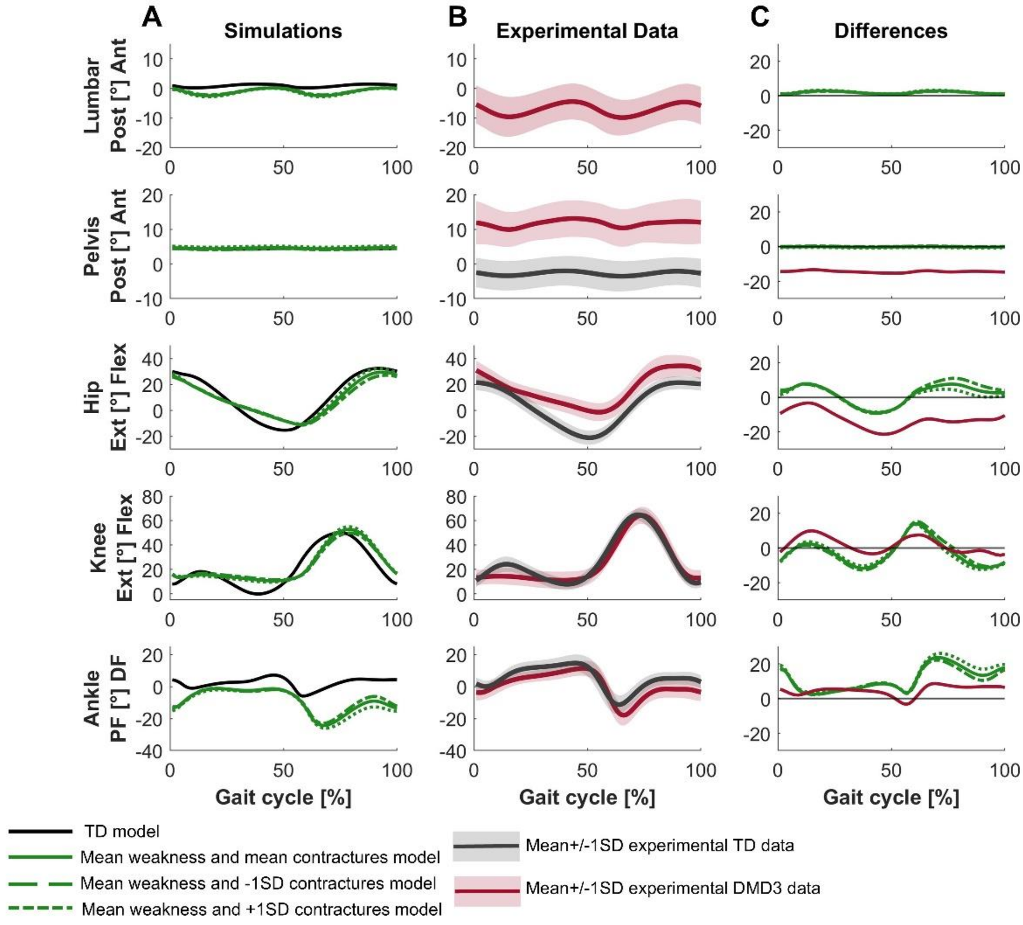

Figure S16: *Sensitivity analysis varying contractures around their mean value by ±1 SD while holding muscle weakness constant at its mean value on sagittal plane kinematics for DMD3.* ***A.*** *Simulated kinematics* ***B.*** *Experimental gait kinematics.* ***C.*** *Differences in kinematics between the TD model and the mean weakness + mean contractures model (solid line), the TD model and the mean weakness with -1SD contractures model (long dotted line), the TD model and the mean weakness with +1SD contractures model (short dotted line), and the experimental TD and DMD3 data (red). Abbreviations: Ant, anterior; DF, dorsiflexion; Ext, extension; Flex, flexion; PF, plantar flexion; Post, posterior; TD, typically developing;*

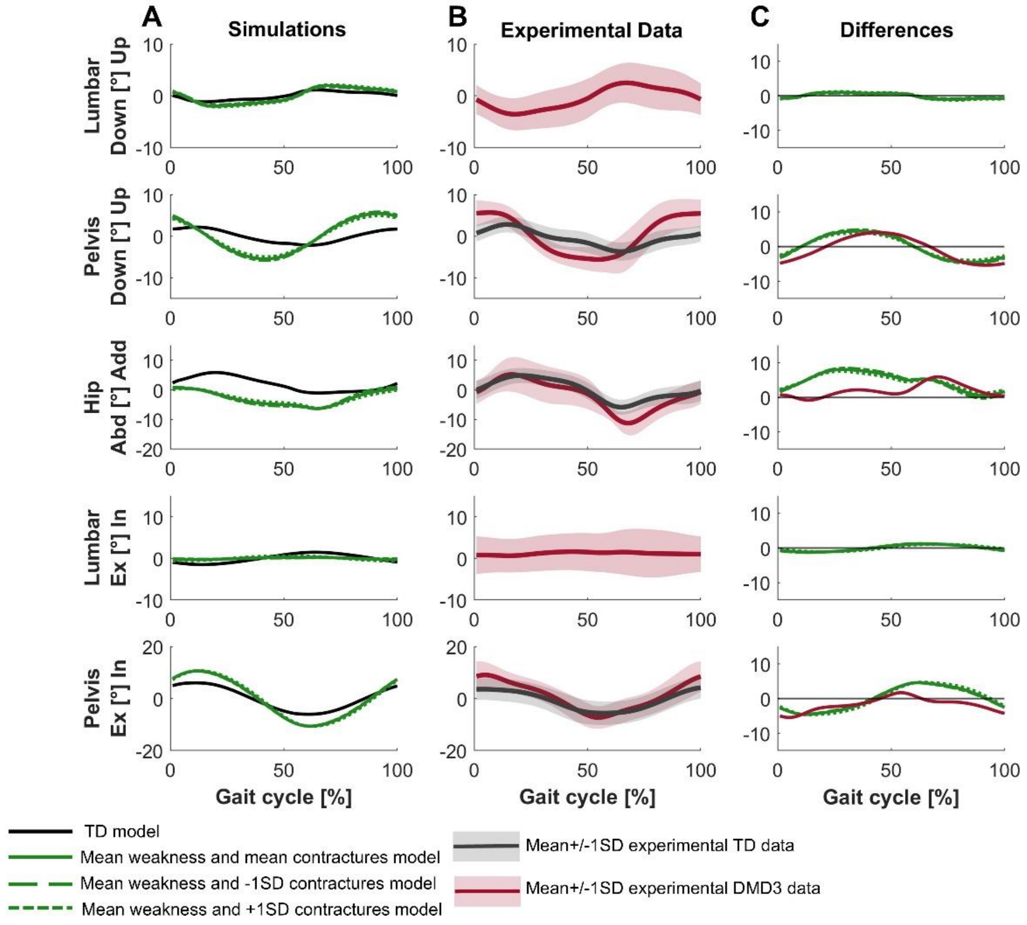

Figure S17: *Sensitivity analysis varying contractures around their mean value by ±1 SD while holding muscle weakness constant at its mean value on frontal and transverse plane kinematics for DMD3.* ***A.*** *Simulated kinematics* ***B.*** *Experimental gait kinematics.* ***C.*** *Differences in kinematics between the TD model and the mean weakness + mean contractures model (solid line), the TD model and the mean weakness with -1SD contractures model (long dotted line), the TD model and the mean weakness with +1SD contractures model (short dotted line), and the experimental TD and DMD3 data (red). Abbreviations: Abd, abduction; Add, adduction; Ex, external; In, internal; TD, typically developing;*

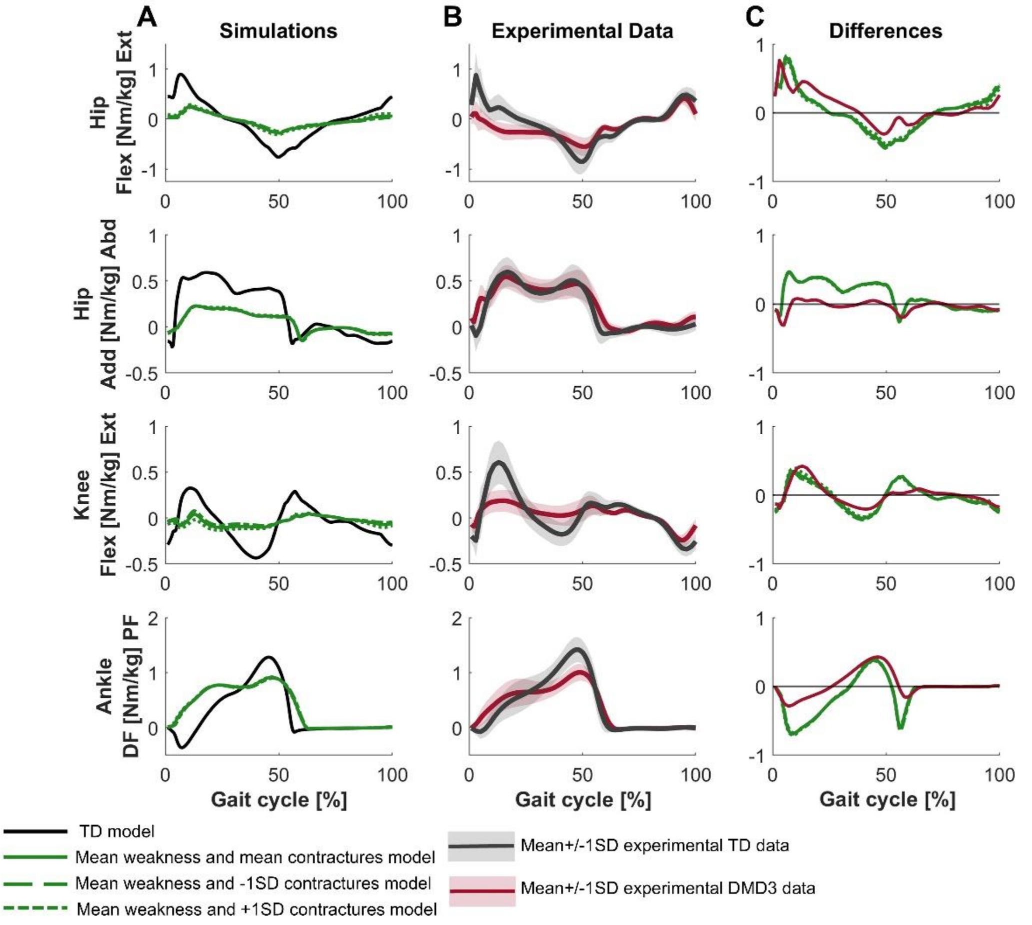

Figure S18: *Sensitivity analysis varying contractures around their mean value by ±1 SD while holding muscle weakness constant at its mean value on kinetics for DMD3.* ***A.*** *Simulated kinetics* ***B.*** *Experimental gait kinetics.* ***C.*** *Differences in kinetics between the TD model and the mean weakness + mean contractures model (solid line), the TD model and the mean weakness with -1SD contractures model (long dotted line), the TD model and the mean weakness with +1SD contractures model (short dotted line), and the experimental TD and DMD3 data (red). Abbreviations: Abd, abduction; Add, adduction; DF, dorsiflexion; Ext, extension; Flex, flexion; PF, plantar flexion; TD, typically developing;*
