# Supplementary material for: Muscle weakness but also contractures contribute to the progressive gait pathology in children with Duchenne muscular dystrophy: a simulation study
Source: J Neuroeng Rehabil. 2025 May 4;22:103. doi: 10.1186/s12984-025-01631-x (PMC12051353; doi:10.1186/s12984-025-01631-x)
Supplement: Supplementary file 5 — Additional file 5. [file 12984_2025_1631_MOESM5_ESM.pdf]

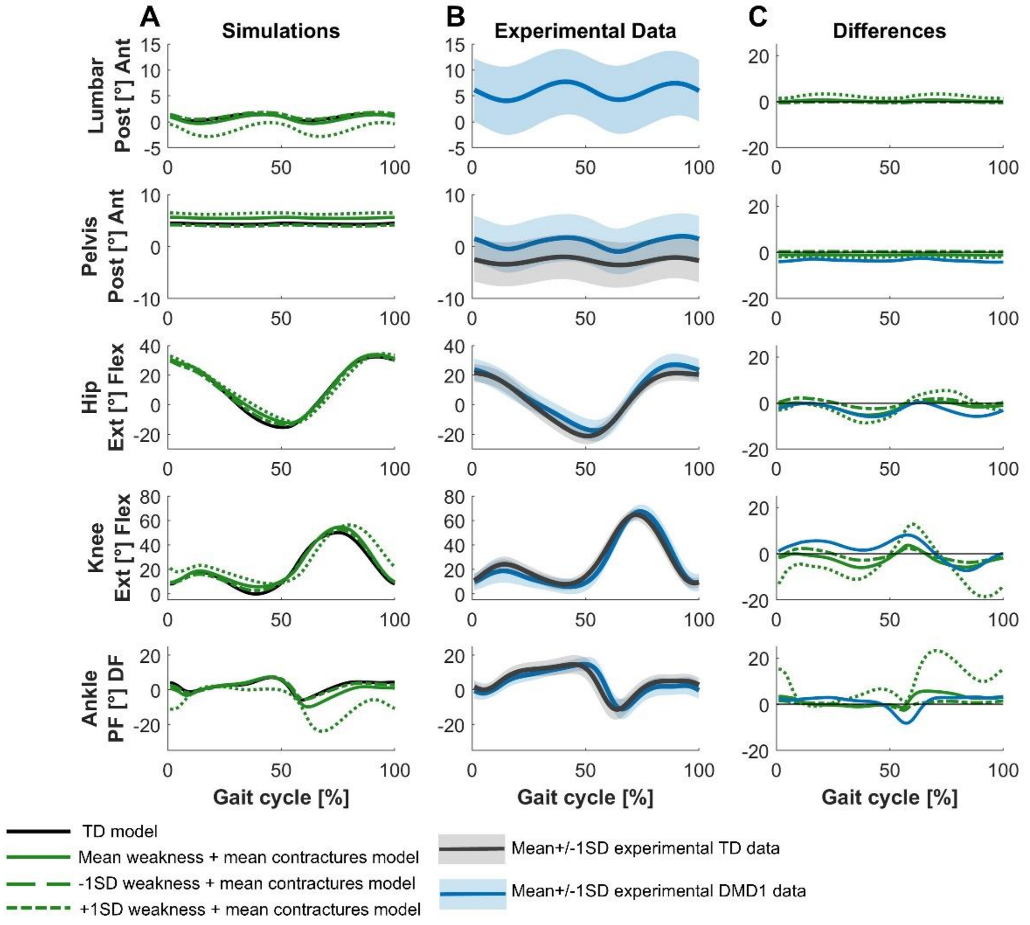


**Figure S19**: *Sensitivity analysis of varying muscle weakness around its mean by ±1 SD while holding contractures constant at their mean value on sagittal plane kinematics for DMD1.* ***A.*** *Simulated kinematics* ***B.*** *Experimental gait kinematics.* ***C.*** *Differences in kinematics between the TD model and the mean weakness + mean contractures model (solid line), the TD model and the -1SD weakness + mean contractures model (long dotted line), the TD model and the +1SD weakness + mean contractures model (short dotted line), and the experimental TD and DMD1 data (blue). Abbreviations: Ant, anterior; DF, dorsiflexion; Ext, extension; Flex, flexion; PF, plantar flexion; Post, posterior; TD, typically developing;*

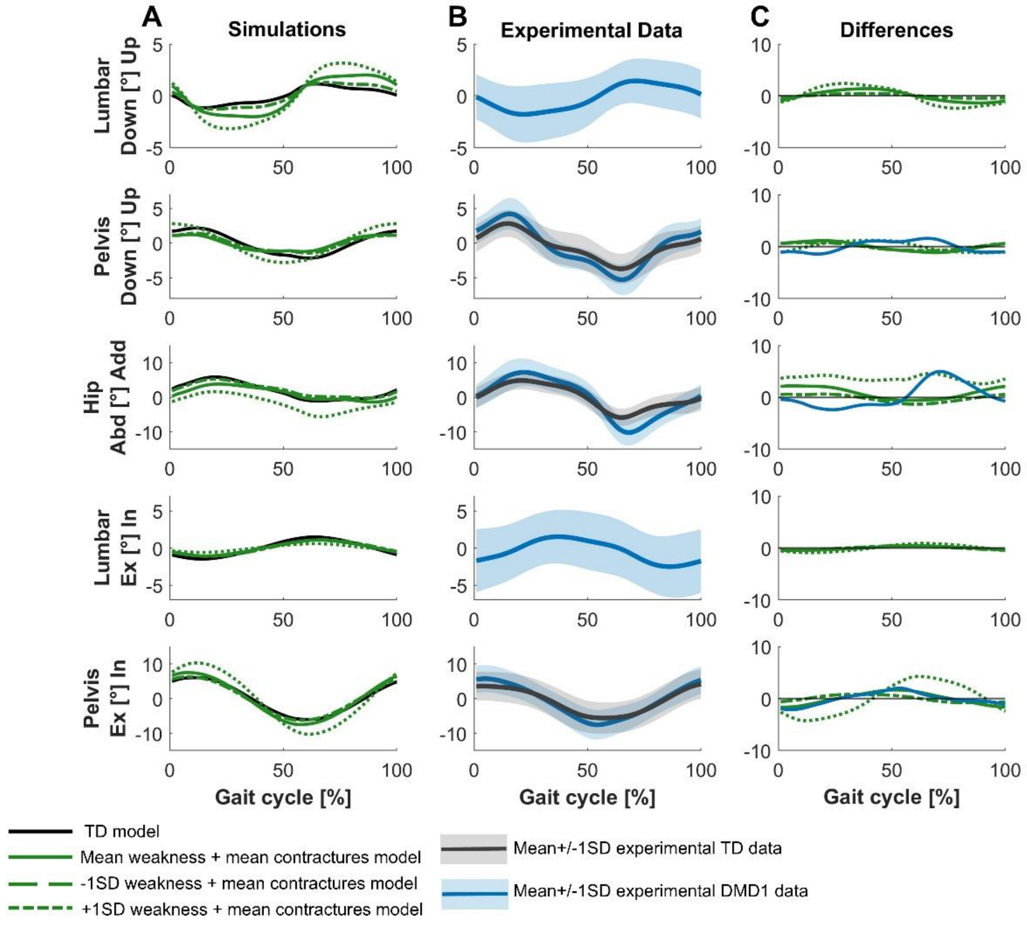


**Figure S20**: *Sensitivity analysis of varying muscle weakness around its mean by ±1 SD while holding contractures constant at their mean value on frontal and transverse plane kinematics for DMD1.* ***A.*** *Simulated kinematics* ***B.*** *Experimental gait kinematics.* ***C.*** *Differences in kinematics between the TD model and the mean weakness + mean contractures model (solid line), the TD model and the -1SD weakness + mean contractures model (long dotted line), the TD model and the +1SD weakness + mean contractures model (short dotted line), and the experimental TD and DMD1 data (blue). Abbreviations: Abd, abduction; Add, adduction; Ex, external; In, internal; TD, typically developing;*

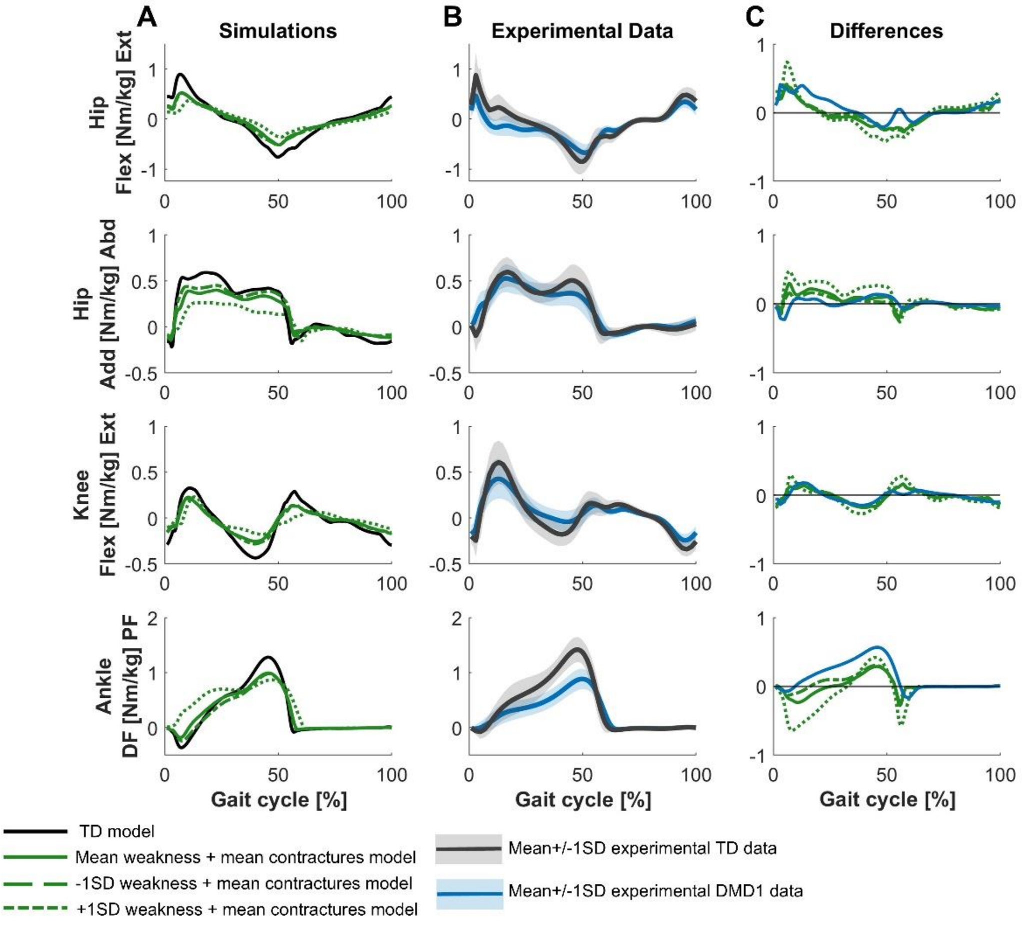


**Figure S21**: *Sensitivity analysis of varying muscle weakness around its mean by ±1 SD while holding contractures constant at their mean value on kinetics for DMD1.* ***A.*** *Simulated kinetics* ***B.*** *Experimental gait kinetics.* ***C.*** *Differences in kinetics between the TD model and the mean weakness + mean contractures model (solid line), the TD model and the -1SD weakness + mean contractures model (long dotted line), the TD model and the +1SD weakness + mean contractures model (short dotted line), and the experimental TD and DMD1 data (blue). Abbreviations: Abd, abduction; Add, adduction; DF, dorsiflexion; Ext, extension; Flex, flexion; PF, plantar flexion; TD, typically developing;*

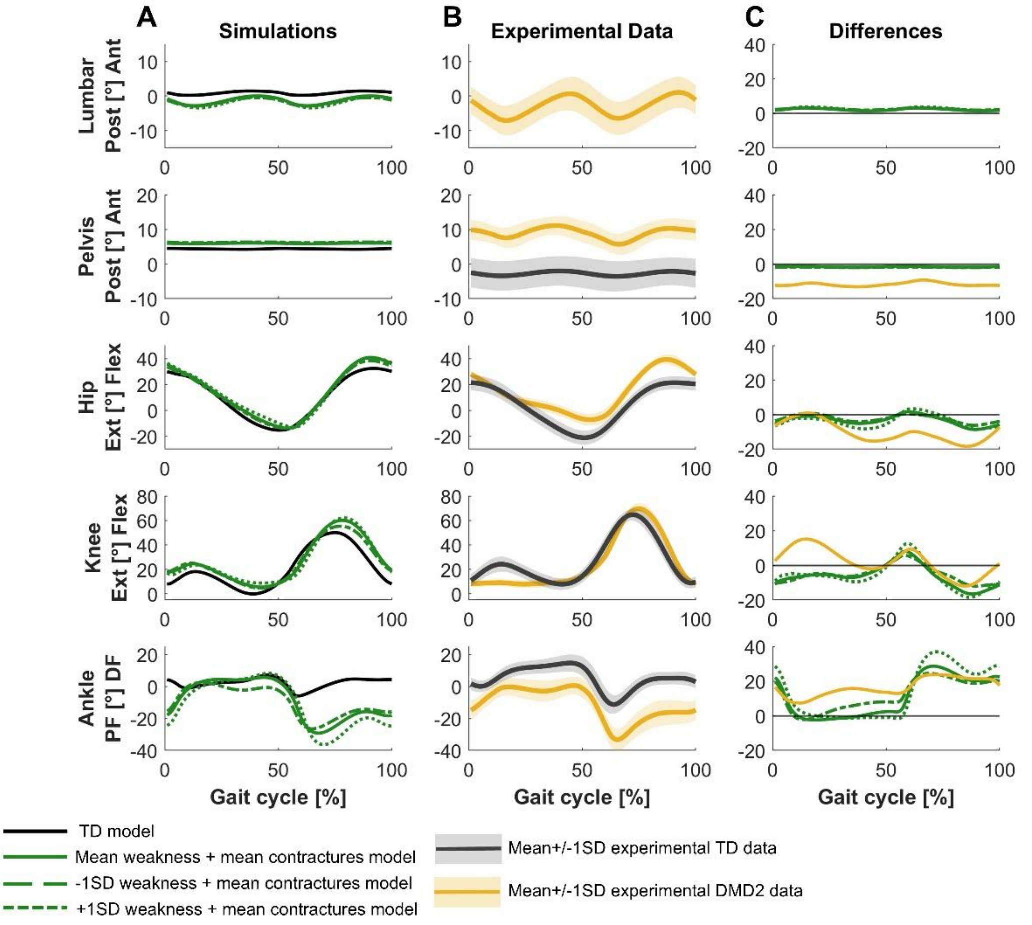


**Figure S22**: *Sensitivity analysis of varying muscle weakness around its mean by ±1 SD while holding contractures constant at their mean value on sagittal plane kinematics for DMD2.* ***A.*** *Simulated kinematics* ***B.*** *Experimental gait kinematics.* ***C.*** *Differences in kinematics between the TD model and the mean weakness + mean contractures model (solid line), the TD model and the -1SD weakness + mean contractures model (long dotted line), the TD model and the +1SD weakness + mean contractures model (short dotted line), and the experimental TD and DMD2 data (yellow). Abbreviations: Ant, anterior; DF, dorsiflexion; Ext, extension; Flex, flexion; PF, plantar flexion; Post, posterior; TD, typically developing;*

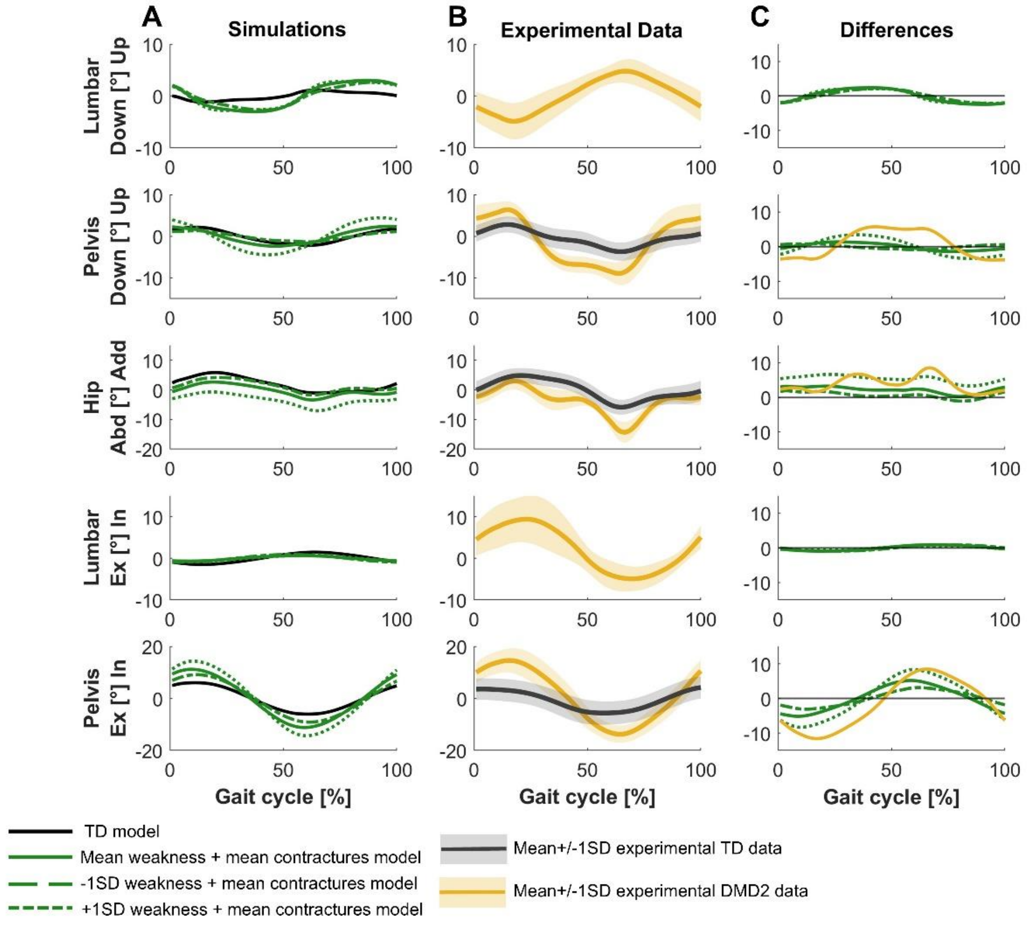


**Figure S23**: *Sensitivity analysis of varying muscle weakness around its mean by ±1 SD while holding contractures constant at their mean value on frontal and transverse plane kinematics for DMD2.* ***A.*** *Simulated kinematics* ***B.*** *Experimental gait kinematics.* ***C.*** *Differences in kinematics between the TD model and the mean weakness + mean contractures model (solid line), the TD model and the -1SD weakness + mean contractures model (long dotted line), the TD model and the +1SD weakness + mean contractures model (short dotted line), and the experimental TD and DMD2 data (yellow). Abbreviations: Abd, abduction; Add, adduction; Ex, external; In, internal; TD, typically developing;*

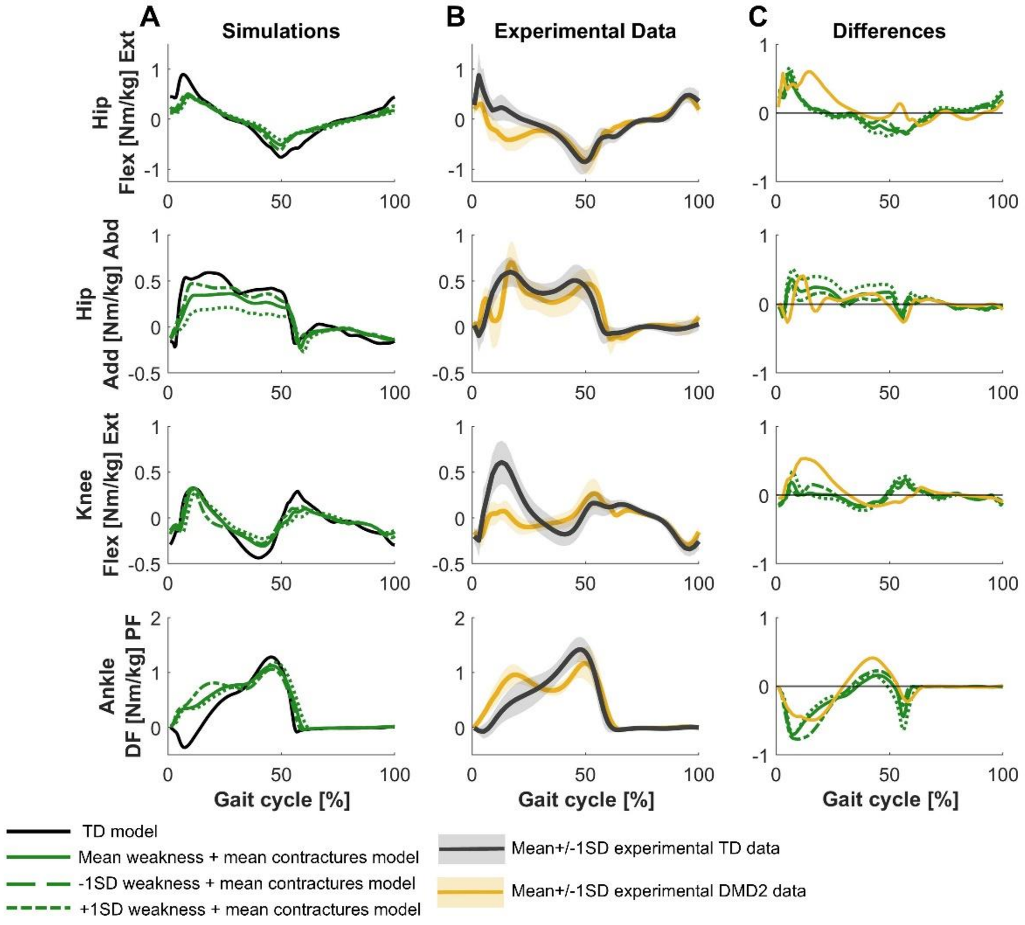


**Figure S24**: *Sensitivity analysis of varying muscle weakness around its mean by ±1 SD while holding contractures constant at their mean value on kinetics for DMD2.* ***A.*** *Simulated kinetics* ***B.*** *Experimental gait kinetics.* ***C.*** *Differences in kinetics between the TD model and the mean weakness + mean contractures model (solid line), the TD model and the -1SD weakness + mean contractures model (long dotted line), the TD model and the +1SD weakness + mean contractures model (short dotted line), and the experimental TD and DMD2 data (yellow). Abbreviations: Abd, abduction; Add, adduction; DF, dorsiflexion; Ext, extension; Flex, flexion; PF, plantar flexion; TD, typically developing;*

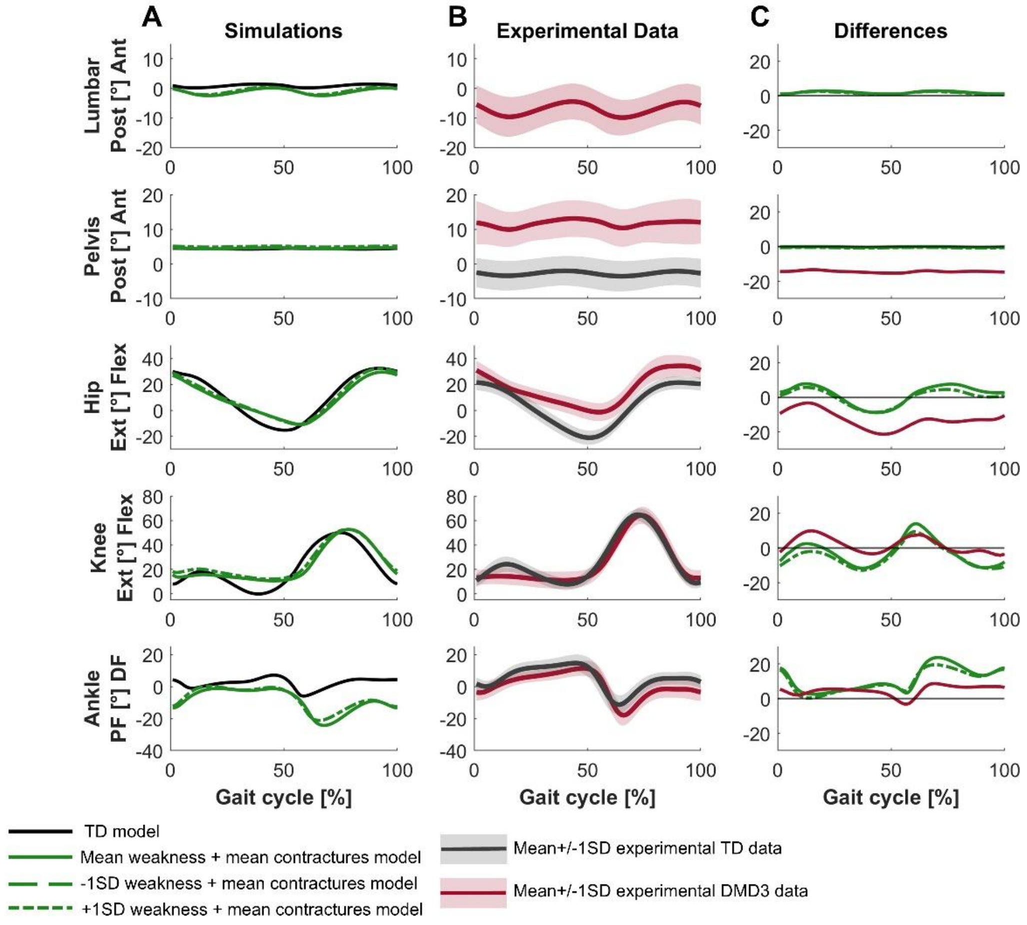


**Figure S25**: *Sensitivity analysis of varying muscle weakness around its mean by ±1 SD while holding contractures constant at their mean value on sagittal plane kinematics for DMD3.* ***A.*** *Simulated kinematics* ***B.*** *Experimental gait kinematics.* ***C.*** *Differences in kinematics between the TD model and the mean weakness + mean contractures model (solid line), the TD model and the -1SD weakness + mean contractures model (long dotted line), and the experimental TD and DMD3 data (red). The simulation based on the +1SD weakness and mean contractures model could not find a feasible solution for DMD3. Abbreviations: Ant, anterior; DF, dorsiflexion; Ext, extension; Flex, flexion; PF, plantar flexion; Post, posterior; TD, typically developing;*

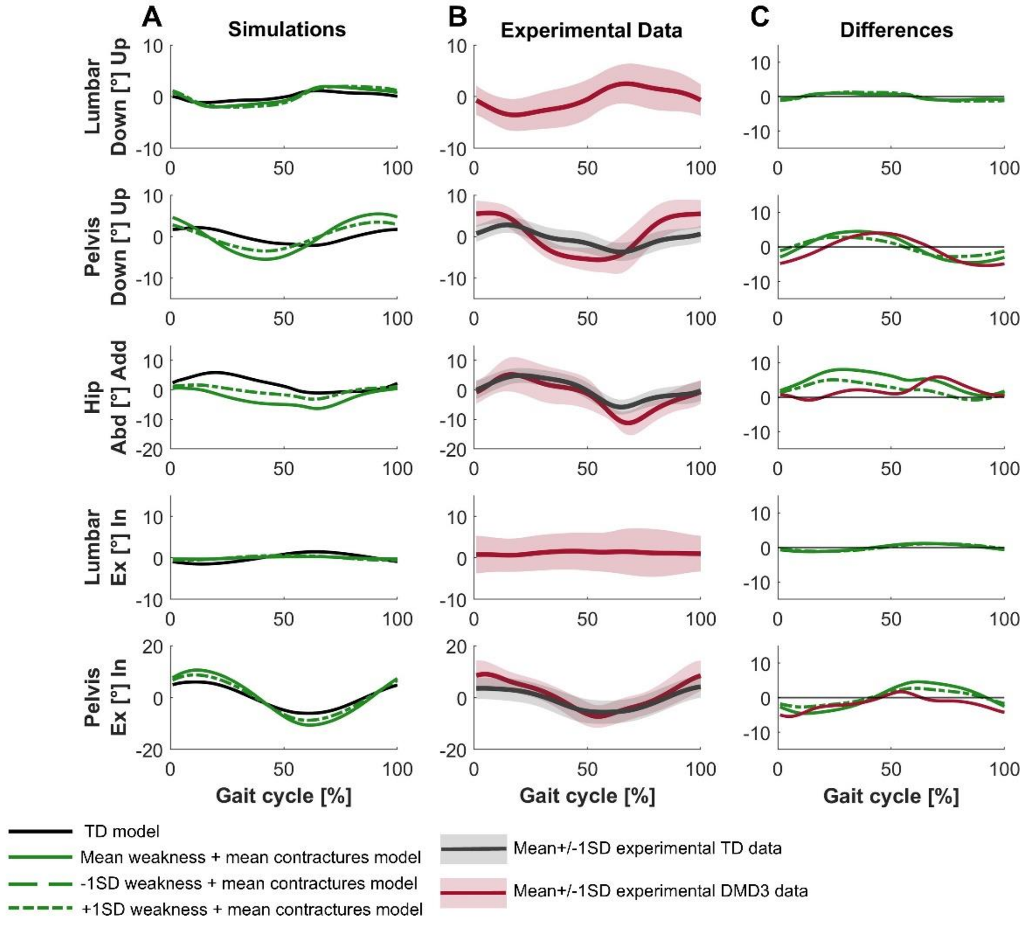


**Figure S26**: *Sensitivity analysis of varying muscle weakness around its mean by ±1 SD while holding contractures constant at their mean value on frontal and transverse plane kinematics for DMD3.* ***A.*** *Simulated kinematics* ***B.*** *Experimental gait kinematics.* ***C.*** *Differences in kinematics between the TD model and the mean weakness + mean contractures model (solid line), the TD model and the -1SD weakness + mean contractures model (long dotted line), and the experimental TD and DMD3 data (red). The simulation based on the +1SD weakness and mean contractures model could not find a feasible solution for DMD3. Abbreviations: Abd, abduction; Add, adduction; Ex, external; In, internal; TD, typically developing;*

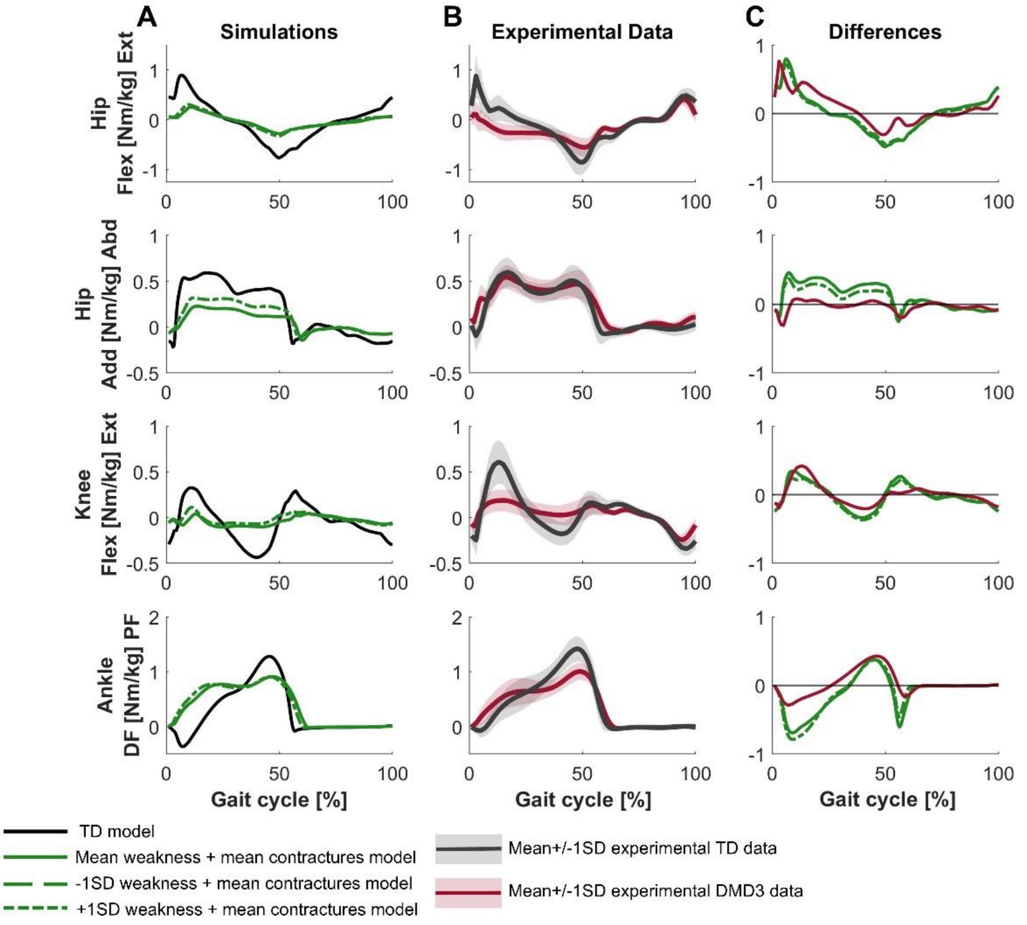


**Figure S27**: *Sensitivity analysis of varying muscle weakness around its mean by ±1 SD while holding contractures constant at their mean value on kinetics for DMD3.* ***A.*** *Simulated kinetics* ***B.*** *Experimental gait kinetics.* ***C.*** *Differences in kinetics between the TD model and the mean weakness + mean contractures model (solid line), the TD model and the -1SD weakness + mean contractures model (long dotted line), and the experimental TD and DMD3 data (red). The simulation based on the +1SD weakness and mean contractures model could not find a feasible solution for DMD3. Abbreviations: Abd, abduction; Add, adduction; DF, dorsiflexion; Ext, extension; Flex, flexion; PF, plantar flexion; TD, typically developing;*
